# Frontal alpha asymmetry and instructed satisfaction-rating changes after price presentation: An exploratory pilot study

**DOI:** 10.1371/journal.pone.0361622

**Published:** 2026-11-02

**Authors:** Passavorn Limmeechokchai, Bu-nga Chaisuwan, Yodchanan Wongsawat, Jetsada Arnin

**Affiliations:** 1 Department of Biomedical Engineering, Faculty of Engineering, Mahidol University, Nakhon Pathom, Thailand; 2 Graduate School of Communication Arts and Management Innovation, National Institute of Development Administration (NIDA), Bangkok, Thailand; Sapienza University of Rome: Universita degli Studi di Roma La Sapienza, ITALY

## Abstract

This exploratory pilot study asked whether frontal alpha asymmetry (FAA) recorded while a price is displayed varies with the satisfaction-rating change a consumer then makes. Twenty-one participants completed two blocks of 30 trials: view a product, give a pre-price rating (1–9), see the price, give a post-price rating, which they were instructed to change except at the scale limits. The change is described by its direction and magnitude (|Δ|, provisionally labeled Decision Momentum), two descriptions of one difference score. FAA was computed from relative alpha power at F3/F4 over 0–3 s after price onset on average-referenced data. Inference rests on 19 participants and 820 trials. The primary pooled test of the direction × magnitude interaction was inconclusive (β=−0.051, 95% CI [−0.111,0.009], *p* = 0.093). An exploratory block-stratified analysis found an interaction in the first block (β=−0.087, 95% CI [−0.152,−0.021], *p* = 0.010), although neither of its two component slopes differed clearly from zero. Support varied across specifications, and five alternative operationalizations of FAA did not reproduce it. The same model applied to the window *before* price onset, where the predictors cannot yet reflect the price, produced an interaction of comparable or larger size pooled and in the second block. In the first block, the direct comparison did not establish a difference between windows, and adjusting for pre-price FAA left the first-block estimate unchanged (*p* = 0.008); in the second block, the two windows did differ (*p* = 0.021), the association being present before the price and not after it. Single-trial classification of change direction showed modest performance with uncertain generalizability (ROC AUC 0.538; participant bootstrap 95% CI [0.476, 0.564]). The association is reported as hypothesis-generating; attributing it specifically to price-related processing is not supported by these data.

## Introduction

### Price evaluation as a core driver of consumer choice

Price evaluation is a central mechanism in consumer decision-making because it is the point at which subjective preference is translated into perceived value-for-money. In everyday online shopping, consumers rarely decide based on product attributes alone; rather, the final judgment emerges when the price is revealed and integrated with an internal reference of expected quality, affordability, and fairness [1,2]. Importantly, this integration process is not purely deliberative. A substantial portion of valuation occurs implicitly, shaped by rapid affective and motivational signals that consumers may not be able to articulate through conventional self-report measures [1,2]. As a result, the observed choice outcome (e.g., buying versus not buying) can be viewed as the endpoint of a dynamic internal update, where pricing information triggers a re-calibration of satisfaction and willingness to engage [2].

### Background and related works

To better capture the implicit dynamics of valuation, neurophysiological tools can complement traditional behavioral approaches by providing time-sensitive indicators of brain activity during the moments when consumers integrate marketing information [1,2]. Electroencephalography (EEG) is particularly well-suited for this purpose because it offers millisecond-level temporal resolution while remaining non-invasive and practical for laboratory paradigms that simulate real-world shopping scenarios [3]. Within EEG-based indices, Frontal Alpha Asymmetry (FAA) has been widely used as a theory-driven marker linked to motivational tendencies described within the approach–withdrawal framework [4–6]. The following subsections review three strands of literature that motivate the design and analytic framework of the present study.

#### From static binary labels to dynamic intensity.

Despite the growing use of EEG markers to predict consumer preference, many neuromarketing paradigms conceptualize the outcome as a binary label—liked versus disliked, or bought versus not bought [3,7,8]. A systematic review of this literature identifies frontal alpha asymmetry as the time-frequency measure most consistently associated with self-reported preference [3]. This binary framing remains common in recent work: hybrid EEG-and-gaze decoders have been trained to classify single trials as “buy” versus “no-buy” [9], and naturalistic online-shopping studies have contrasted EEG responses to products that were bought against those that were discarded [10]. This framing is useful for classification, but it overlooks a key behavioral reality: decisions are not equally decisive. Two trials may both end in a purchase, yet differ drastically in how strongly the consumer’s evaluation shifted after seeing the price. Likewise, a non-purchase can reflect either mild hesitation or a sharp negative reappraisal. Collapsing these heterogeneous processes into a single binary label may dilute neural effects in single-trial analyses, where signal variability is high—a possibility that motivates the present design rather than an established finding. Therefore, a framework that accounts for the intensity of the behavioral update may be necessary to reveal when and why frontal EEG markers become more informative. We note that, although we draw on this purchase-framed literature for background, the present study measures the magnitude of a post-price satisfaction-rating update rather than an actual purchase choice.

#### Frontal Alpha Asymmetry (FAA) as a state-sensitive marker.

A second consideration concerns how FAA itself should be interpreted across repeated measurements. Reviews of the frontal asymmetry literature emphasize that this index can reflect both relatively stable, trait-like individual differences and more transient, state-dependent fluctuations, and that careful operational choices—montage and reference selection, preprocessing, and quality control—are critical for distinguishing between the two and for drawing interpretable inferences [5,6]. Classic psychometric work further shows that resting anterior EEG asymmetry, while exhibiting meaningful temporal stability, can still vary with context and measurement conditions [5,11]. Importantly, FAA is not restricted to slowly varying resting-state measurement: event-related designs have demonstrated that brief, sufficiently salient stimuli can themselves elicit immediate, phasic shifts in frontal asymmetry, rather than requiring several minutes of continuous exposure [12]. This state-sensitive property is directly relevant to the present paradigm, in which the price-reveal moment constitutes a brief, motivationally salient event nested within a longer recording session; it provides the theoretical basis for examining both FAA during the price-display window (FAA_post_) and its relationship to a pre-stimulus baseline (FAA_pre_) within the same trial, as well as for treating between-session shifts in tonic FAA as a separate, baseline-related phenomenon to be assessed directly rather than assumed away.

#### Moderating factors in pricing research.

A third consideration concerns factors, beyond the price-related decision itself, that can shape frontal EEG measures during product evaluation. Trial-by-trial preference for the product being viewed is one such factor: EEG power over frontal and centro-parietal sites has been shown to differentiate “like” from “dislike” decisions during consumer evaluation [13]. Likewise, frontal asymmetry itself has been linked to multiple, only partly overlapping facets of price-related evaluative updating—including categorical purchase decisions [14]. Findings across studies are not uniform, however, and depend on the frequency band examined: in a willingness-to-pay paradigm, prefrontal asymmetry in the gamma band (25–40 Hz) predicted subsequent willingness-to-pay responses (*p* = 0.004), whereas asymmetry in the alpha band (8–13 Hz) did not (*p* = 0.19) [15]. Frontal asymmetry should therefore not be treated as a single measure with a single meaning across bands and tasks. These observations suggest that any attempt to isolate a price-related signature must account for such factors rather than treating decision-related variance as homogeneous across trials. Accordingly, the present analytic framework incorporates trial-level product liking (RatingProduct) as a covariate alongside the direction and magnitude of the rating change.

### Quantifying the size of the rating change

To address this limitation, we quantify the *magnitude* of the rating change, |Δ|, alongside its direction, and refer to this magnitude by the provisional operational label *Decision Momentum*. The rationale is that two trials ending in the same direction may differ in how far the evaluation moved, and that a framework distinguishing them may reveal when frontal EEG measures are more detectable than a binary label allows. We state three qualifications at the outset. First, in this paradigm the change is made under explicit instruction (Materials and methods), so |Δ| is the size of an instructed revision, not evidence that price altered the evaluation. Second, direction and magnitude are two descriptions of one difference score rather than separately measured constructs, which determines how the model must be read (Data Categorization). Third, a difference between two ratings does not establish decisiveness, motivational intensity, or the temporal persistence that “momentum” ordinarily implies; no external criterion was collected against which the quantity could be validated. We therefore use *rating-change magnitude* as the descriptive term throughout and treat the label as provisional.

### Objectives of the study

This study examines whether frontal alpha asymmetry (FAA) recorded at F3/F4 while a price is displayed varies with the satisfaction-rating change a participant then makes. Accordingly, the study is guided by the following objectives:

1. To describe the direction and magnitude of satisfaction-rating changes made after price presentation, noting that these changes were made under instruction and that purchase choices were not measured.
2. To characterize frontal alpha asymmetry at F3/F4 in the window during which the price is displayed, and to relate it to the direction and magnitude of the rating change. A further question—whether any such association is specific to that window rather than already present before price onset—was added during revision and is therefore exploratory rather than part of the original design.
3. To test whether the direction of a rating change can be classified from FAA at the single-trial level, and whether classification depends on the magnitude of the change.

Given the sample size, all objectives are pursued in an exploratory, feasibility-oriented spirit rather than as confirmatory tests.

## Materials and methods

### Participants

A total of 21 participants (aged 18–27 years) were recruited from Mahidol University; recruitment and data collection were carried out between 26/03/2025 and 27/04/2025 (DD/MM/YYYY). All participants reported prior experience with online shopping and provided written informed consent prior to the experimental sessions. EEG recordings were conducted at the Faculty of Engineering, Mahidol University, Thailand. The recruited sample comprised 8 female and 13 male participants (the final analyzed sample of 19 comprised 7 female and 12 male; see Results).

#### Inclusion criteria.

1. Age 18–27 years.
2. Self-reported annual personal income below THB 360,000 (approximately USD 11,250, based on an exchange rate of 1 USD = 32 THB).
3. Prior experience with online shopping.

#### Exclusion criteria.

1. History of neurological disorders (e.g., cerebrovascular disease, movement disorders, multiple sclerosis, or prior brain surgery).
2. History of epilepsy or seizures.
3. Current or previously diagnosed psychiatric conditions that may affect cognition or emotional regulation.

Participation was voluntary, and no personally identifiable information was retained in the analysis dataset.

#### How eligibility was assessed.

All criteria were assessed by participant self-report on a paper screening form administered in person by the first author at recruitment, at least 24 hours before the first session and in the same contact in which the participant information sheet was provided and written informed consent was obtained (see Ethical Considerations). The form’s items restated the inclusion and exclusion criteria listed above; age was recorded as a value, prior online-shopping experience and each exclusion criterion as yes/no responses, and annual personal income only as meeting or not meeting the stated ceiling. No clinical interview, medical record, or documentary verification was used, and no diagnostic instrument was administered; the neurological and psychiatric exclusions therefore rest on self-report alone, and undisclosed or undiagnosed conditions cannot be excluded. The screening items are reproduced in S1 Appendix.

#### Income.

The annual personal income ceiling (below THB 360,000) was applied as an eligibility criterion at recruitment and was recorded only as a yes/no eligibility check; neither an exact income nor an income band was collected. Income could therefore not be entered as a covariate or examined as a moderator, and the sample is by construction range-restricted on income. Because purchasing power plausibly shapes how far a displayed price moves a satisfaction rating, this is a substantive limitation rather than a technicality: clustering by participant accounts for repeated measures but does not adjust for unmeasured income or affordability (see Limitations). Together with the university-based recruitment and the 18–27 year age range, the criterion yields a relatively young, lower-to-middle-income sample, which should be taken into account when generalizing the findings to older or higher-income consumer populations.

### Product type selection and stimuli design

Consumer goods are often categorized into convenience, shopping, specialty, and unsought products, which differ in the typical level of deliberation required before purchase. In the present EEG paradigm, the key design constraint was to elicit a measurable evaluative process within a short, trial-based time window.

Accordingly, this study focused on **shopping products**, which typically involve a moderate degree of comparison (e.g., perceived quality relative to price) while remaining feasible for rapid, repeated decisions in a laboratory task. In contrast, high-involvement or specialty purchases may require extended deliberation that cannot be realistically modeled in a few-second trial, whereas convenience goods may be decided with minimal conscious evaluation, potentially reducing sensitivity to pricing-related neural modulation.

#### Stimulus selection criteria.

Product stimuli were selected using the following criteria:

1. Items commonly available on Thai e-commerce platforms and recognizable to local consumers;
2. Non-perishable consumer goods suitable for individual purchase (i.e., excluding services, subscriptions, and consumables);
3. Clear and unambiguous product depiction in a single image (frontal or standard catalog view) with minimal background clutter;
4. No explicit promotional overlays (e.g., discount badges, free-gift banners) to avoid confounding salience effects;
5. Prices were selected to remain within a plausible, non-extreme range for each category to maintain a comparable level of deliberation; in the final stimulus set, prices ranged from THB 11–12,232 overall (median 399; IQR 178–890), with category-specific ranges of bags (THB 59–6,390), electrical appliances (THB 59–12,232), and furniture (THB 11–3,486).

A total of 60 product images were used as stimuli: 20 bags, 20 electrical appliances, and 20 furniture items. Images and price information were sourced from the two most widely used e-commerce platforms in Thailand, Shopee Thailand and Lazada Thailand and prepared for standardized on-screen presentation (e.g., fixed image size and resolution, consistent background, and uniform placement) to enhance ecological validity and participant familiarity.

### Experimental procedure and trial design

The experimental workflow and trial structure are summarized in Fig 1. The experiment was conducted in two sessions, each comprising 30 trials, separated by a 5-minute rest period. Both sessions followed an identical procedure but used different stimulus sets to reduce repetition effects. Each session presented 30 distinct products with no overlap between sessions, balanced by category (10 bags, 10 electrical appliances, and 10 furniture items per session). Product category was therefore matched across sessions; however, the specific items (and their prices and images) differed, and the session order was fixed rather than counterbalanced—all participants viewed stimulus set A in Session 1 and set B in Session 2.

**Fig 1:**
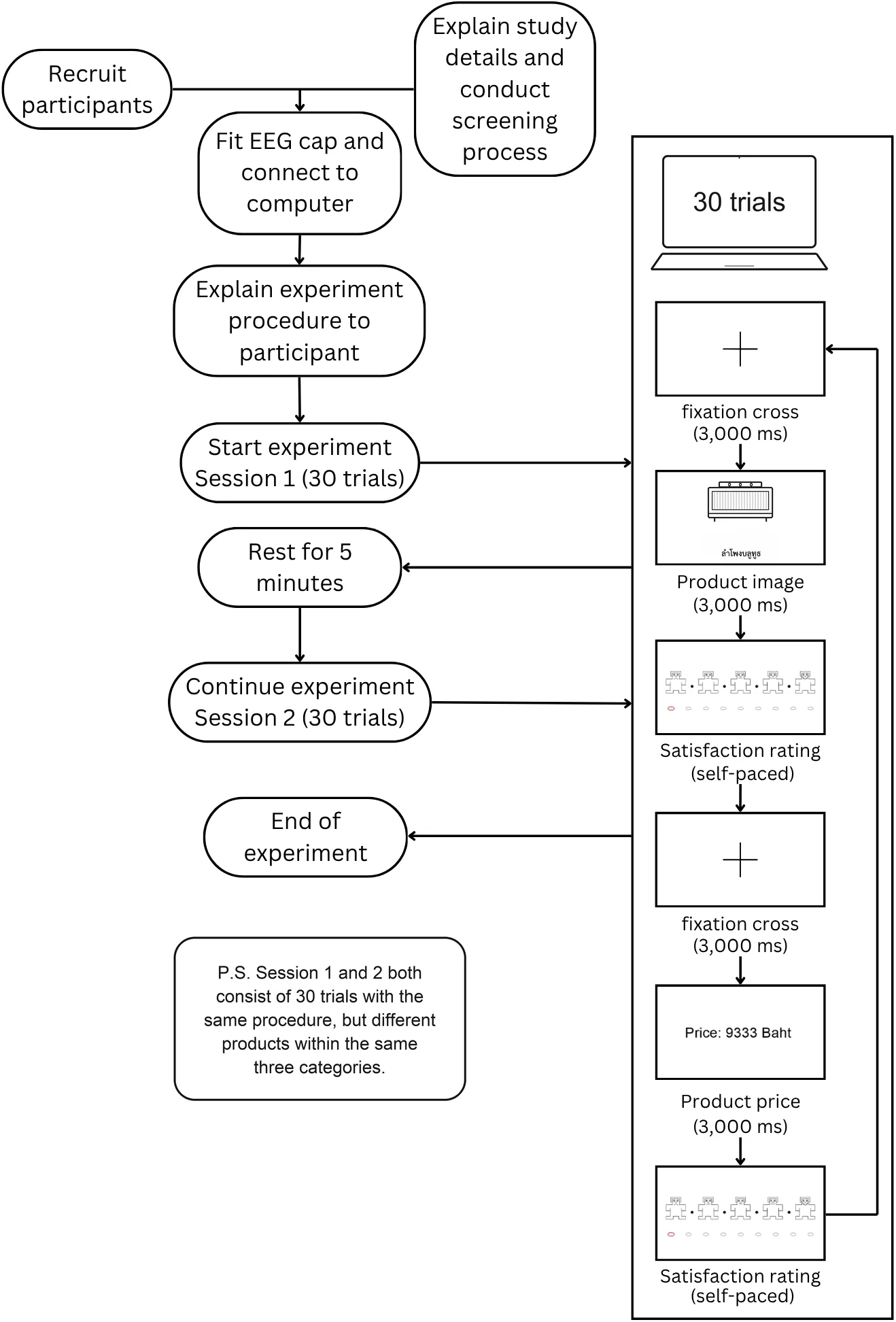
Experimental flow and trial sequence. All elements of this figure were drawn by the authors. Left: study workflow from recruitment and screening to EEG setup and completion of two sessions (Session 1 and Session 2), separated by a 5-minute rest. Sessions used different products drawn from the same three categories. Right: structure of a single trial (fixation cross, product image, pre-price rating, fixation cross, price display, and post-price rating). Rating responses were self-paced (no time limit). The product shown in the “product image” panel is a schematic drawing prepared by the authors for illustration; the stimuli actually presented were photographs obtained from Thai e-commerce listings, which are third-party copyrighted material and are therefore not reproduced here. The price shown is likewise illustrative.

**Why Session is modeled, and what its coefficient can and cannot mean:** Matching on product category does not make the two blocks equivalent: they presented different products, different prices, and different images, and they occurred in a fixed order. The two sets also differed in price level: set A had a median displayed price of THB 618 (IQR 202–1,156) against THB 339 (IQR 175–755) for set B, so the products participants saw in the first block were about 1.8 times more expensive at the median. A rank test at 30 items per set does not reach significance (Mann–Whitney *U* = 522, *p* = 0.29), but that does not make the descriptive difference uncertain, and it does not remove the confounding of block with stimulus set. Session was therefore included as a covariate to absorb average differences between the two sequential blocks rather than to test a hypothesis about time. Because block order and stimulus set are fully confounded, its coefficient represents a combined session/stimulus-set contrast and cannot isolate an effect of elapsed time, fatigue, habituation, or the products themselves. We report it in those terms throughout and return to the point when interpreting the between-block difference.

#### Trial sequence.

Each trial simulated an online product-evaluation scenario and followed the same sequence:

1. **Fixation cross (3,000 ms):** participants fixated on a central cross to stabilize attention.
2. **Product image (3,000 ms):** participants viewed the product for initial evaluation.
3. **Rating 1 (Pre-price; self-paced):** participants rated their satisfaction on a 9-point scale (1 = least satisfied, 9 = most satisfied).
4. **Fixation cross (3,000 ms):** attentional reset before price presentation.
5. **Price display (3,000 ms):** the product price was shown on screen.
6. **Rating 2 (Post-price; self-paced):** participants provided a second satisfaction rating after viewing the price.

To ensure that the post-price rating reflected price integration, participants were instructed that the post-price rating must differ from the pre-price rating, except when the pre-price rating was at the scale limits (1 or 9), where further decreases or increases were not possible.

Visual stimuli and rating responses were presented and collected using PsychoPy. EEG signals and event markers were recorded concurrently and synchronized in real time via OpenViBE and LabRecorder.

### EEG data acquisition

EEG was recorded using a **g.USBamp biosignal amplifier** and a **g.GAMMASYS active electrode system** (g.tec medical engineering GmbH, Austria) at a sampling rate of 512 Hz. Electrodes were placed according to the international 10–20 system. The acquisition montage included Fp1, Fp2, F7, F8, F3, Fz, F4, C3, Cz, C4, P3, Pz, P4, AF3 and AF4. The ground electrode was positioned at AFz, and the reference electrode was attached to a single earlobe (A1 or A2), selected per participant based on impedance and signal quality checks. Electrode impedances were kept below 5 kΩ and monitored using the amplifier’s impedance check prior to recording. Because the online single-earlobe reference is a lateralized site that could in principle bias a frontal asymmetry measure, all data were re-referenced offline to an average reference before FAA computation. Thus, FAA was not computed from the online single-earlobe reference. However, because average referencing remains montage-dependent, reference effects remain a limitation of the present 15-channel setup. The recording arrangement is illustrated in Fig 2B. That panel is a drawing rather than a photograph: it was produced by the authors with the assistance of a generative image tool and then edited, and the person depicted is a generic figure who is not a study participant and whose face is obscured. No data, results, or measurements in this manuscript were generated or altered by such tools.

**Fig 2:**
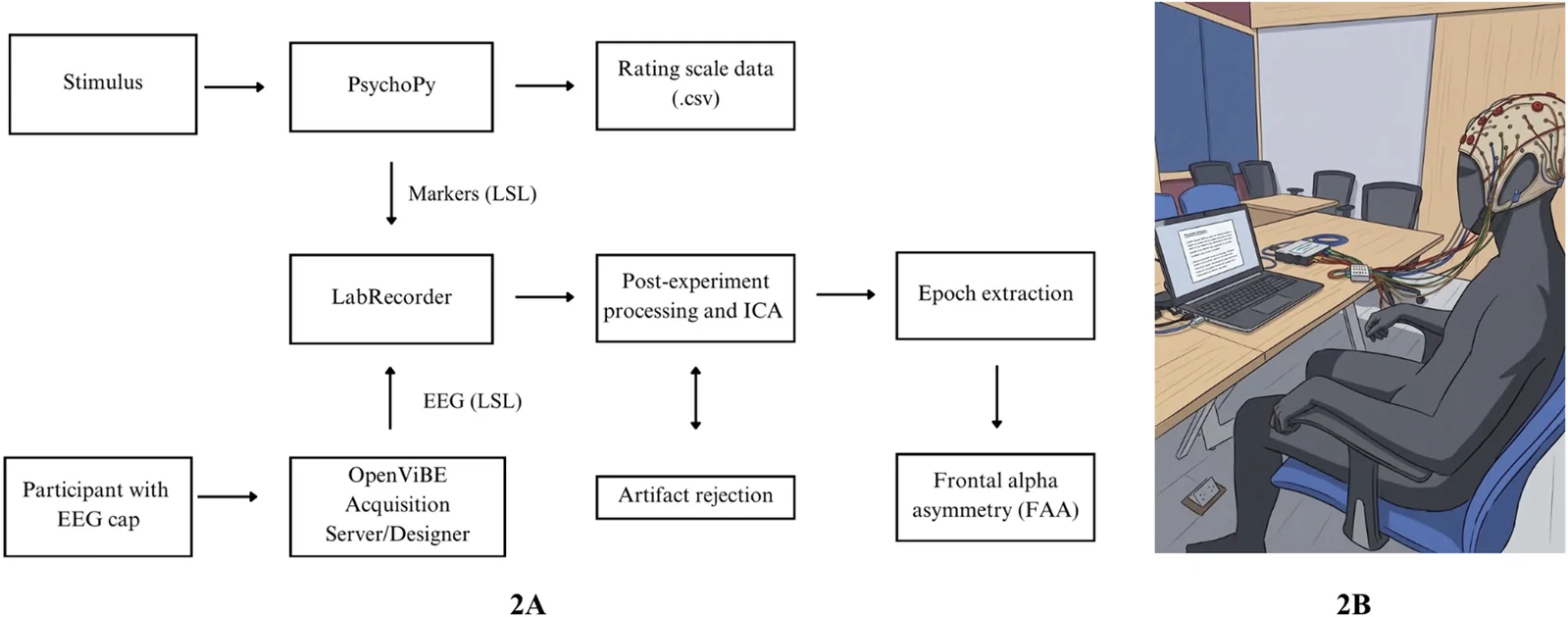
EEG acquisition and analysis overview. All elements of panel A were drawn by the authors. **(A)** Signal path and analysis pipeline: stimuli and rating scales were presented in PsychoPy, which wrote the behavioral ratings and sent event markers over LSL; EEG was acquired through the OpenViBE acquisition server and recorded together with the markers by LabRecorder; offline processing comprised filtering, ICA, artifact rejection, epoch extraction around price onset, and computation of frontal alpha asymmetry. **(B)** Illustration of the recording arrangement: a seated participant wearing the EEG cap, facing the monitor on which stimuli were presented, with the acquisition wiring and grounding indicated. The panel is an illustration prepared by the authors, not a photograph; the depicted person is a generic figure and does not portray the likeness of any study participant, and the face is obscured.

Stimulus presentation and behavioral ratings were implemented in PsychoPy. EEG acquisition and event synchronization were handled using the OpenViBE Acquisition Server/Designer together with LabRecorder, which stored time-synchronized EEG and event-marker streams in XDF format for offline processing. Behavioral responses (pre-price and post-price satisfaction ratings on a 1–9 scale) were recorded on each trial and exported for analysis.

**ICA preparation and segment-level quality control (adapted)** The acquisition and preprocessing reporting in this section follows established community recommendations for reproducible EEG/MEG research [16,17]. The ICA preparation and segment-level quality control procedures were adapted from a commonly used preprocessing strategy in frontal alpha asymmetry research [18]. Briefly, the continuous EEG used for downstream analyses was band-pass filtered from 0.3 to 45 Hz, followed by a 50 Hz notch (line-noise) filter. All filters were zero-phase FIR filters (Hamming window; MNE-Python defaults): the 0.3–45 Hz band-pass used lower/upper transition bandwidths of 0.3/11.25 Hz (−6 dB cutoffs at 0.15/50.6 Hz, filter length 5,633 samples ≈11.0 s), and the notch used a 49–51 Hz band-stop. For ICA decomposition, a separate copy of the continuous data was high-pass filtered at 1 Hz (zero-phase FIR, lower transition bandwidth 1 Hz, length 1,691 samples ≈3.3 s) and segmented into non-overlapping 1-s epochs. Following this strategy, ICA-training epochs were screened by amplitude rejection (epochs exceeding 1000 μV peak-to-peak in any channel), and a “20% channel rule” was applied as a safeguard: any channel flagged as bad in more than 20% of the ICA-training epochs would be excluded from both the ICA-copy and the original recording. In the present dataset, the amplitude criterion removed only a small number of ICA-training epochs and no channel met the 20% exclusion threshold; consequently, no channels were excluded. ICA (extended Infomax algorithm; 15 components, equal to the number of EEG channels; maximum 500 iterations) was then fitted on the ICA-copy and the resulting unmixing weights were applied to the original continuous data.

Artifact-related components were identified by visual inspection of component time courses, scalp maps, and spectra, and the selected components (predominantly ocular components reflecting blinks and horizontal eye movements, recognized by their characteristic frontopolar topography and low-frequency time course) were removed prior to epoching. Across the 42 recordings, a single component was removed in 36 recordings and two components in 6 recordings (48 components in total). After IC removal, bad channels (if any) were interpolated and the data were re-referenced to the average reference.

While the original implementation used EEGLAB-based utilities, the present study followed the same conceptual preprocessing logic using an MNE-based pipeline [19,20]. All analyses were conducted in Python 3.12 using MNE-Python 1.12, statsmodels 0.14, scikit-learn 1.9, NumPy 2.1, pandas 3.0, and SciPy 1.18. Stimulus presentation and behavioral logging used PsychoPy 2024.2.4, and EEG acquisition/synchronization used the OpenViBE Acquisition Server 2.2.0 (32-bit) and OpenViBE Designer 3.6.0 (64-bit) with LabRecorder 1.16.2.

**Task-epoch artifact rejection (analysis set)** For the main analysis, price-locked epochs were extracted from the ICA-cleaned continuous data using event markers (show_price1), with an epoch window of 0–3 s and no baseline correction. By design, the price was displayed for 3,000 ms before the post-price rating scale appeared; across all 1,260 recorded trials the rating-scale onset marker followed the price onset by 3.01–3.10 s (mean 3.03 s), and no trial presented the response interface within the 0–3 s analysis window. Because participants could not enter the self-paced rating until the scale appeared, motor preparation and response execution for the rating necessarily occurred after the analyzed window, limiting motor contamination of the FAA estimate. Epoch-level artifact rejection was then applied using a 150 μV peak-to-peak threshold (epochs exceeding 150 μV in any channel were dropped). This criterion removed 301 of the 1,260 price-locked epochs (23.9%); the rejection rate varied widely across recordings (from 0 to all 30 trials). One participant whose recordings were almost entirely rejected, together with a second participant who did not meet the minimum trial-per-class requirement, was subsequently removed by the participant-level screening (see Results). Artifact-rejection rates did not differ by session (*p* = 1.00) or Valence (*p* = 0.94); the association with rating-change magnitude was non-significant but showed a weak trend (*p* = 0.075), which we note as a minor caveat. To enable tonic/baseline analyses without changing the trial set, an additional epoch set was reconstructed with a wider window (−1 to 3 s), but epoch rejection was still determined only from the task window (0–3 s) using the same 150 μV threshold; rejected trial indices were then removed from the full (−1 to 3 s) epochs.

### Data categorization: Direction and magnitude of the rating change

For each trial, participants provided a pre-price satisfaction rating and a post-price satisfaction rating on a 1–9 scale. The rating shift was defined as $\Delta ={\text{Rating}}_{post}-{\text{Rating}}_{pre}$. Trials were categorized into two groups based on the direction of the shift (Valence): **Positive Shift** (Δ>0) and **Negative Shift** (Δ<0). To handle scale-limit cases where a change is impossible, trials with 9→9 were treated as Positive Shift and trials with 1→1 were treated as Negative Shift, consistent with the study instruction that post-price ratings should differ unless constrained by the scale boundary. Decision Momentum was defined as |Δ| for trials with a non-zero rating change; scale-boundary no-change trials (9→9, 1→1; *N* = 117) were excluded from the primary momentum models to avoid assigning a magnitude to a zero change, retained in the session-only FAA analyses and in the sensitivity classification that assigns them a direction, and added back under a minimum-magnitude (|Δ|=1) coding as a sensitivity analysis (see Results). Because the task instructed participants to provide a post-price rating that differed from the pre-price rating except at scale boundaries (see Trial sequence), this magnitude should be interpreted as the size of an *instructed* post-price rating revision rather than a spontaneous re-evaluation; it does not directly measure purchase intention, real-world buying behavior, or spontaneous decision confidence. Throughout the manuscript we therefore use *rating-change magnitude* as the descriptive term for |Δ| and treat *Decision Momentum* as a provisional operational label for the same quantity, not as an independently validated construct: a difference between two ratings does not by itself establish decisiveness, motivational intensity, or the temporal persistence that the word “momentum” ordinarily implies.

**Direction and magnitude are two descriptions of one observed change:** Writing $\Delta =R_{post}-R_{pre}$, M=|Δ| and V=𝕀(Δ<0), the signed change satisfies


$$\begin{array}{c} \Delta =M\;(1-2V), \end{array}$$

so that span{1,Δ,M}⊂span{1,V,M,V·M}. The model used here (Valence, magnitude, and their interaction) is therefore not a model of two independently measured psychological variables; it is a two-slope, piecewise-linear fit of the signed rating change, with one additional parameter representing a discontinuity at Δ=0. Its interaction term should accordingly be read as “the magnitude–FAA slope differs between the positive and negative sides of zero,” which is the interpretation we adopt throughout, and we report the two implied slopes directly alongside it (Results, Signed change score and direction-specific slopes). A further consequence of the same identity,


$$\begin{array}{c} R_{post}=R_{pre}+M-2VM, \end{array}$$

is that entering both ratings together with *M* and V·M produces an exactly linearly dependent design matrix; this, rather than merely correlated predictors, is why the dual-rating specification reported below does not converge.

### Feature extraction and statistical analysis

Frontal alpha asymmetry (FAA) was computed from the frontal electrode pair F3/F4 during the price display epoch (0–3 s). The F3/F4 pair was specified as the primary site in the study’s analysis plan, written before data collection began, on theoretical grounds, as it is the canonical mid-frontal montage in the approach–withdrawal FAA literature [4–6]; exploratory results for the other available frontal pairs (F7/F8, AF3/AF4) are reported separately (see Results). Power spectral density was estimated using Welch’s method [21] with a 512-sample (1 s) Hamming window, no overlap, and a resulting frequency resolution of 1 Hz; segments were averaged within each epoch. The post-price window (0–3 s) therefore comprised three 1-s segments, whereas the pre-price baseline (FAA_pre_, −1 to 0 s) comprised a single 1-s segment and thus yields a coarser, single-segment estimate—a limitation we note explicitly when interpreting baseline-referenced quantities. The baseline window fell entirely within the 3-s fixation period preceding the price display. How much signal the filtering may carry across that boundary was not quantified (see Results), so we describe the window by where it falls rather than as free of residual price processing. Relative alpha power was defined as the ratio of alpha-band power (8–13 Hz) to total power (1–40 Hz); the relative (rather than absolute) formulation was used to normalize for broadband, non-asymmetry differences in overall power across participants and sessions. FAA was computed as:


$$\begin{array}{c} \text{FAA}=\ln({\text{RelAlpha}}_{F4})-\ln({\text{RelAlpha}}_{F3}). \end{array}$$

We note that this is not the conventional frontal-asymmetry metric. The usual measure is the difference in log alpha *power* (or power density) between homologous sites, without a broadband denominator [5,6]; the relative formulation used here additionally divides by total power, which is equivalent to subtracting a broadband asymmetry term (see Results). It was chosen to reduce non-asymmetry differences in overall power across participants and blocks, and the conventional absolute-alpha version is reported alongside it as a sensitivity analysis.

To evaluate whether FAA varied with the direction and magnitude of the rating change, pooled regression models were fitted with **Valence**, **Decision Momentum**, their interaction, and covariates (RatingProduct and Session). To account for within-subject dependence, robust inference was performed using subject-clustered standard errors and generalized estimating equations with an exchangeable working correlation structure [22, 23]. As a sensitivity analysis, the same model specification (excluding Session) was also fitted separately within each session.

#### Analysis hierarchy and inference.

The Core Decision–Momentum Model (CDM; FAA ∼ Valence × Momentum + RatingProduct + Session) fitted to the pooled primary set was designated as the primary analysis in the original submission, before the analyses added at revision, with GEE as the primary estimator and cluster-robust OLS as a sensitivity estimator. Every other analysis reported here is secondary, sensitivity or exploratory, and is labeled as such where it appears: the session-stratified models, the signed-change and direction-specific slope reparameterizations, the baseline (FAA_pre_, *d*FAA, posterior-alpha) analyses, the three-way Valence × magnitude × Session term, the sensitivity package, the pre-price window analyses, and the single-trial classification. The stratified models in particular were not pre-specified and do not confer primary status on either session. Because *p*-values are not adjusted for multiple comparisons, all results beyond the primary test are hypothesis-generating.

Given inference from 19 participant clusters, the primary and exploratory interaction estimates are additionally reported under bias-reduced (CR2) cluster-robust covariance with Satterthwaite degrees of freedom and under a wild cluster bootstrap; a linear mixed-effects model with a participant random intercept and a within-participant permutation test are reported alongside them. We treat the spread across these procedures as the range of uncertainty rather than as evidence that any one of them is definitive, since none is free of small-sample assumptions. The permutation test permutes only relabelings that the 1–9 rating scale admits, because an unconstrained shuffle of shift direction can generate combinations of direction, magnitude and pre-price rating that cannot occur (see Results).

#### Implementation.

Analyses were run in Python 3.12.4 with numpy 2.1.3, scipy 1.18.0, pandas 3.0.3, statsmodels 0.14.6, patsy 1.0.2, scikit-learn 1.9.0 and MNE-Python 1.12.1. GEE models used statsmodels with a Gaussian family and an exchangeable working correlation, grouped by participant; cluster-robust least squares used the same grouping. The bias-reduced (CR2) covariance with Satterthwaite degrees of freedom and the wild cluster bootstrap (Rademacher weights, null imposed, *B* = 9,999, seed 0) were implemented directly and applied to the least-squares fit. The direction permutation used *B* = 5,000 within-participant permutations (seed 0); the single-trial classification used *B* = 1,000 label permutations per threshold (seed 100 + threshold; one-sided, testing performance above chance) and *B* = 2,000 participant bootstrap resamples (seed 7), in which every copy of a resampled participant kept that participant’s group label so that all copies were held out together, with the classifier refitted inside every resample. Analysis code is also available for reproducibility (see the data availability statement).

The precision of the primary test is reported as an approximate sensitivity calculation based on the observed standard error, holding that standard error fixed (two-sided α=0.05, 80% power); it does not depend on the observed estimate. To address the possibility that magnitude merely tracks price extremity, a price-deviation covariate—each item’s displayed price, log-transformed and standardized within its product category—was added to the model.

For visualization, we produced “hybrid” plots combining model-implied trends and binned descriptive summaries. Model lines were computed from the session-stratified CDM with RatingProduct fixed at its sample mean. Binned points represent mean FAA_post_ within rating-change magnitude ranges (1–2, 3–4, 5–8), with error bars indicating 95% cluster bootstrap intervals obtained by resampling participants with replacement. Numbers next to points indicate the number of trials contributing to each bin.

### Ethical considerations

This study was approved by the **Mahidol University Central Institutional Review Board (MU-CIRB)**, Faculty of Engineering site, under Protocol No. MU-CIRB 2025/023.1401 (expedited review; approved March 25, 2025). All procedures were conducted in accordance with the Declaration of Helsinki and with applicable national and institutional guidelines for research involving human participants.

**All participants provided written informed consent** at least 24 hours prior to participation, after receiving the participant information sheet and having the opportunity to ask questions; no consent was waived. The consent process was voluntary and non-coercive, and participants were given adequate time to consider their involvement. The study was classified as minimal risk and involved non-invasive EEG recording and brief behavioral tasks. Data were anonymized at the point of export and securely stored. Under the original approved protocol, study data will be retained for two years following completion of the study and then destroyed; requests for access can therefore be made to MU-CIRB within that period (see the data availability statement). The study team adhered to MU-CIRB reporting requirements, including progress reporting and notification of protocol amendments when applicable.

## Results

### Analysis population and behavioral rating changes

The full retained dataset comprised repeated single-trial observations from *N* = 19 participants (*N*_obs_ = 937 trials). The trial flow was as follows: of the 1,260 possible trials (21 participants × 60 trials), 301 (23.9%) were removed by the 150 μV peak-to-peak artifact criterion in the 0–3 s task window, leaving 959 artifact-free trials. A participant-level screening rule—requiring at least three Positive Shift and three Negative Shift trials in *each* of the two sessions—then excluded two participants (one whose recordings were almost entirely artifact-rejected and one who failed the per-class minimum), removing a further 22 trials and yielding the final set of 937 trials from 19 participants. The primary magnitude models and the principal classification analysis included the 820 trials with an observed non-zero rating change, the remaining 117 being scale-boundary no-change trials (9→9, 1→1). Session-only FAA analyses, which need no directional label, retained all 937 trials. Classification using assigned directions for the 117 boundary trials was conducted separately as a sensitivity analysis (see Data Categorization). Direction was approximately balanced. Among the 820 trials with an observed change, 445 were positive and 375 negative; across the 937-trial retained set, which assigns a direction to the boundary trials, the corresponding counts are 458 and 479.

**Size of the instructed rating change:** All inferential models involving magnitude were fitted to the primary momentum set (*N*_obs_ = 820 trials from *N* = 19 participants), and we report the behavioral descriptives for that set: mean |Δ|=2.00 scale points (*SD* = 1.38), median 2, IQR 1–2, range 1–8. This corresponds to 25.0% of the 8-point range over which a rating could move. The distribution was concentrated at small changes: 49.4% of trials were one-point changes, 27.8% two-point, and 22.8% three points or larger; the number of participants contributing trials fell from 19 at |Δ|=1–2 to 11 at |Δ|=5 and 1 at |Δ|=8, so estimates in the upper tail rest on few participants as well as few trials. We note that the value of 1.87 reported previously is the mean of the 937-trial retained set, in which the 117 scale-boundary trials carry an artificial minimum-magnitude code of |Δ|=1 rather than an observed change; the two sets are therefore not interchangeable, and we use 2.00 (*SD* = 1.38) for the models reported here. We do not characterize this magnitude as large or small, as no behavioral benchmark exists for a satisfaction-rating revision of a given size on this scale.

Because an artifact-rejected trial still has a valid pair of ratings, we also computed the same descriptives from the behavioral records alone, independently of every EEG exclusion. Across all non-boundary trials of the 19 analyzed participants (*n* = 999), mean |Δ| was 2.06, compared with 2.00 in the EEG-qualified primary set—a difference of −0.06 scale points—indicating that EEG-based exclusion did not appreciably shift the behavioral distribution being modeled. Compliance with the change instruction was complete: across all 1,260 recorded trials there were no non-boundary trials with Δ=0, and the 148 zero-change trials (117 of which survived artifact rejection) were all at a scale limit (9→9 or 1→1). Because a change was instructed except at those limits, |Δ| describes the size of an instructed revision rather than whether price altered the evaluation at all.

### Primary pooled analysis

The primary test, designated as such in the original submission, was the Valence × magnitude interaction in the pooled CDM (primary momentum set, *N*_obs_ = 820 trials from *N* = 19 participants). **It did not provide conclusive evidence for the interaction**: GEE β=−0.0510, *SE* = 0.0304, 95% CI [−0.1106,0.0086], *p* = 0.0932 (cluster-robust OLS β=−0.0562, *p* = 0.0661; Table 1). The small-sample corrections, which are applied to the corresponding least-squares estimate (cluster-robust OLS *p* = 0.066), move it further from significance rather than closer (CR2 *p* = 0.118; wild cluster bootstrap *p* = 0.127; see Sensitivity Analyses). The interval does not, however, license the opposite claim: an approximate sensitivity calculation from the observed standard error (*SE* = 0.030), assuming a two-sided test at α=0.05 and 80% power and holding that standard error fixed, returns |β|≈0.085; the exploratory Session 1 estimate of −0.087 lies inside the pooled interval. The pooled test is thus inconclusive about effects of that size rather than evidence against them. (This figure is the effect size that a two-sided test at α=0.05 would detect with 80% power given the observed standard error, holding that standard error fixed; it is an approximation, not a post-hoc power calculation.)

**Table 1:** Robust pooled regression model predicting FAA_post_ (0–3 s after price onset). The Core Decision–Momentum Model (CDM) includes Valence, Momentum, RatingProduct, and Session. Primary estimator: GEE (exchangeable). Sensitivity: OLS with cluster-robust standard errors by participant.

Model	Term	Coef	SE	*p*	95% CI
**CDM:** FAA~Valence×Momentum+RatingProduct+Session (*N*_obs_ = 820, *N*_subj_ = 19)					
GEE	Intercept	−0.1013	0.0656	0.1225	(−0.2299, 0.0273)
GEE	Valence (Negative)^a^	0.1352	0.0670	**0.0435**	(0.0039, 0.2665)
GEE	Session 2^b^	−0.0815	0.0470	0.0832	(−0.1737, 0.0107)
GEE	Momentum (|Δ|)	0.0511	0.0263	0.0524	(−0.0005, 0.1026)
GEE	Valence (Negative)×Momentum	−0.0510	0.0304	0.0932	(−0.1106, 0.0086)
GEE	RatingProduct	0.0178	0.0095	0.0616	(−0.0009, 0.0365)
OLS (cluster)	Intercept	−0.1180	0.0709	0.0960	(−0.2570, 0.0210)
OLS (cluster)	Valence (Negative)^a^	0.1430	0.0698	**0.0405**	(0.0062, 0.2798)
OLS (cluster)	Session 2^b^	−0.0832	0.0485	0.0861	(−0.1782, 0.0118)
OLS (cluster)	Momentum (|Δ|)	0.0584	0.0288	**0.0428**	(0.0019, 0.1150)
OLS (cluster)	Valence (Negative)×Momentum	−0.0562	0.0306	0.0661	(−0.1162, 0.0037)
OLS (cluster)	RatingProduct	0.0176	0.0100	0.0786	(−0.0020, 0.0373)

Primary estimator: GEE with exchangeable working correlation to account for within-participant repeated trials. Sensitivity estimator: OLS with cluster-robust standard errors by participant.

^a^Valence is dummy-coded with Positive Shift as the reference category.

^b^Session is dummy-coded with Session 1 as the reference category.

The remaining pooled terms are reported with intervals and without trend language. The magnitude term was β=0.0511, 95% CI [−0.0005,0.1026], *p* = 0.052 under GEE (β=0.0584, *p* = 0.043 under cluster-robust OLS). The Valence term was β=0.1352, 95% CI [0.0039, 0.2665], *p* = 0.044, but this is the direction contrast evaluated at |Δ|=0, a value absent from the analysis set; at the observed minimum it is β=0.084 (*p* = 0.035) and at the sample mean β=0.033, 95% CI [−0.013,0.080], *p* = 0.16 (see Signed Change Score and Direction-Specific Slopes). The session/stimulus-set contrast in FAA_post_ was β=−0.0815, 95% CI [−0.1737,0.0107], *p* = 0.083 (Session 2 lower); the between-block difference is estimated more directly by the analyses in Table 3. Product liking was β=0.0178, 95% CI [−0.0009,0.0365], *p* = 0.062: an interval consistent with anything from no association to a small positive one, which we therefore do not describe as a contribution to FAA variability. A planned Dual-Rating Extended Model including both RatingProduct and RatingPrice did not converge in the primary momentum-analysis set (*N* = 820). This is a deterministic consequence of the coding rather than a numerical accident: because $R_{post}=R_{pre}+M-2VM$ (Data Categorization), entering both ratings alongside magnitude and its interaction with direction yields an exactly linearly dependent design matrix—a redundant parameterization, not merely correlated predictors. The specification is therefore not identifiable in principle, was not used for inference, and the Core Decision–Momentum Model was retained as the primary specification.

### Signed change score and direction-specific slopes

Because Valence and magnitude are two descriptions of the same observed change (Data Categorization), we report the model in the two additional forms that make its content explicit.

**Signed change score:** Entering the signed change Δ as a single linear predictor—the simplest alternative specification—showed no association with FAA_post_, either pooled (β=−0.0033, 95% CI [−0.0148,0.0081], *p* = 0.57) or in Session 1 (β=−0.0028, 95% CI [−0.0210,0.0153], *p* = 0.76). The specification did not provide clear evidence of a linear association between signed rating change and FAA. It is also not the form the rest of the analysis examines: the pattern under study is a difference in slope between the two sides of zero, which a single linear term in Δ cannot represent.

**Does the two-slope structure earn its parameters?** Four nested specifications were compared on the primary set (S3 Table): a linear term in Δ (AIC 1216.6); the slope free to differ by direction (AIC 1214.2); that model plus a discontinuity at Δ=0, which is the Core Decision–Momentum Model (AIC 1211.6); and signed change entered as a factor with the 16 levels that occur (±1 to ±8; zero does not occur because no-change trials are excluded), which did not improve on the two-slope form (AIC 1223.8). These information criteria are computed by ordinary least squares and do not account for the repeated-measures structure, so we treat them as descriptive.

Two of the three steps differ by a single parameter and can therefore be tested on the coefficient itself, with the estimators used throughout. Allowing the slope to differ by direction gave β=0.0219, 95% CI [−0.0228,0.0665], GEE *p* = 0.34 (wild cluster bootstrap on the least-squares fit, *p* = 0.46), and adding the discontinuity gave β=0.1352, 95% CI [0.0039, 0.2665], GEE *p* = 0.044 (bootstrap *p* = 0.071). Once participant dependence is accounted for, therefore, neither step is clearly supported, and the piecewise structure should be read as the parameterization we adopt rather than as a form the data select. The discontinuity term is in any case evaluated at |Δ|=0, a value that does not occur in this set.

**Separate slopes:** The slopes implied by the CDM, per one-point increase in |Δ| and with delta-method intervals from the cluster-robust covariance, are given in Table 2. In Session 1 the Positive-shift slope was + 0.073 (95% CI [−0.002,0.147]) and the Negative-shift slope −0.014 (95% CI [−0.084,0.057]). Neither slope is individually distinguishable from zero; what the analysis supports is that they differ from one another ($\beta_{diff}=-0.087$, 95% CI [−0.152,−0.021], *p* = 0.010), and this difference is the exploratory Session 1 interaction reported above, restated in interpretable units.

**Table 2:** Direction-specific slopes and the signed change score. All quantities are linear combinations of the Core Decision–Momentum Model coefficients (GEE, exchangeable working correlation), with delta-method standard errors from the cluster-robust covariance matrix; they therefore cannot be reconstructed by adding the confidence limits of individual coefficients. Slopes are per one-point increase in |Δ|. The signed-change rows come from a separate model in which Δ enters as a single linear predictor.

Set	Quantity	Est.	SE	95% CI	*p*
**Pooled** (*N*_obs_ = 820, *N*_subj_ = 19)					
Pooled	Slope, Positive shift	+ 0.0511	0.0263	(−0.0005, 0.1026)	0.0524
Pooled	Slope, Negative shift	+ 0.0000	0.0258	(−0.0505, 0.0505)	0.9998
Pooled	Difference between slopes^a^	−0.0510	0.0304	(−0.1106, 0.0086)	0.0932
Pooled	Signed change Δ (linear)^b^	−0.0033	0.0059	(−0.0148, 0.0081)	0.5686
**Session 1** (*N*_obs_ = 421, *N*_subj_ = 19)					
S1	Slope, Positive shift	+ 0.0729	0.0380	(−0.0017, 0.1474)	0.0553
S1	Slope, Negative shift	−0.0139	0.0359	(−0.0843, 0.0565)	0.6996
S1	Difference between slopes^a^	−0.0867	0.0335	(−0.1524, −0.0211)	**0.0096**
S1	Signed change Δ (linear)^b^	−0.0028	0.0092	(−0.0210, 0.0153)	0.7580
**Session 2** (*N*_obs_ = 399, *N*_subj_ = 19)					
S2	Slope, Positive shift	+ 0.0283	0.0277	(−0.0259, 0.0825)	0.3065
S2	Slope, Negative shift	+ 0.0156	0.0278	(−0.0389, 0.0701)	0.5738
S2	Difference between slopes^a^	−0.0127	0.0413	(−0.0936, 0.0683)	0.7593
S2	Signed change Δ (linear)^b^	−0.0020	0.0119	(−0.0254, 0.0214)	0.8653

^a^The difference between the two slopes is the Valence × magnitude interaction term of the CDM, restated in slope units; it is the same quantity reported in Tables 1 and 4.

^b^Model: FAA~Δ+RatingProduct (+ Session when pooled), fitted on the same trials.

**Magnitude of the predicted contrast:** The coefficients reported throughout are unstandardized: for FAA outcomes they are in FAA units (a difference in log relative alpha power) per unit of the predictor, which for |Δ| is one rating-scale point. A value below .25 is therefore not small by that fact and cannot be judged against conventions for standardized coefficients, so we express the estimate as a contrast instead. Moving from |Δ|=1 to |Δ|=5 on Positive-shift trials corresponds to a predicted change in FAA_post_ of + 0.29 in Session 1 (95% CI [−0.01,0.59]; 0.57 SD of the trial-level FAA distribution, whose SD is 0.51) and −0.06 on Negative-shift trials (95% CI [−0.34,0.23]). *The interval for the positive-shift contrast includes zero*, and the contrast is estimated from sparse data at its upper end: only 7 Session 1 Positive-shift trials, from 2 participants, have |Δ|≥5 (24 trials from 11 participants on the Negative side). Predicted values at large |Δ| in Fig 3 therefore rest on sparse support and are uncertain; they should not be read as an estimated effect size for high-magnitude trials.

**Fig 3:**
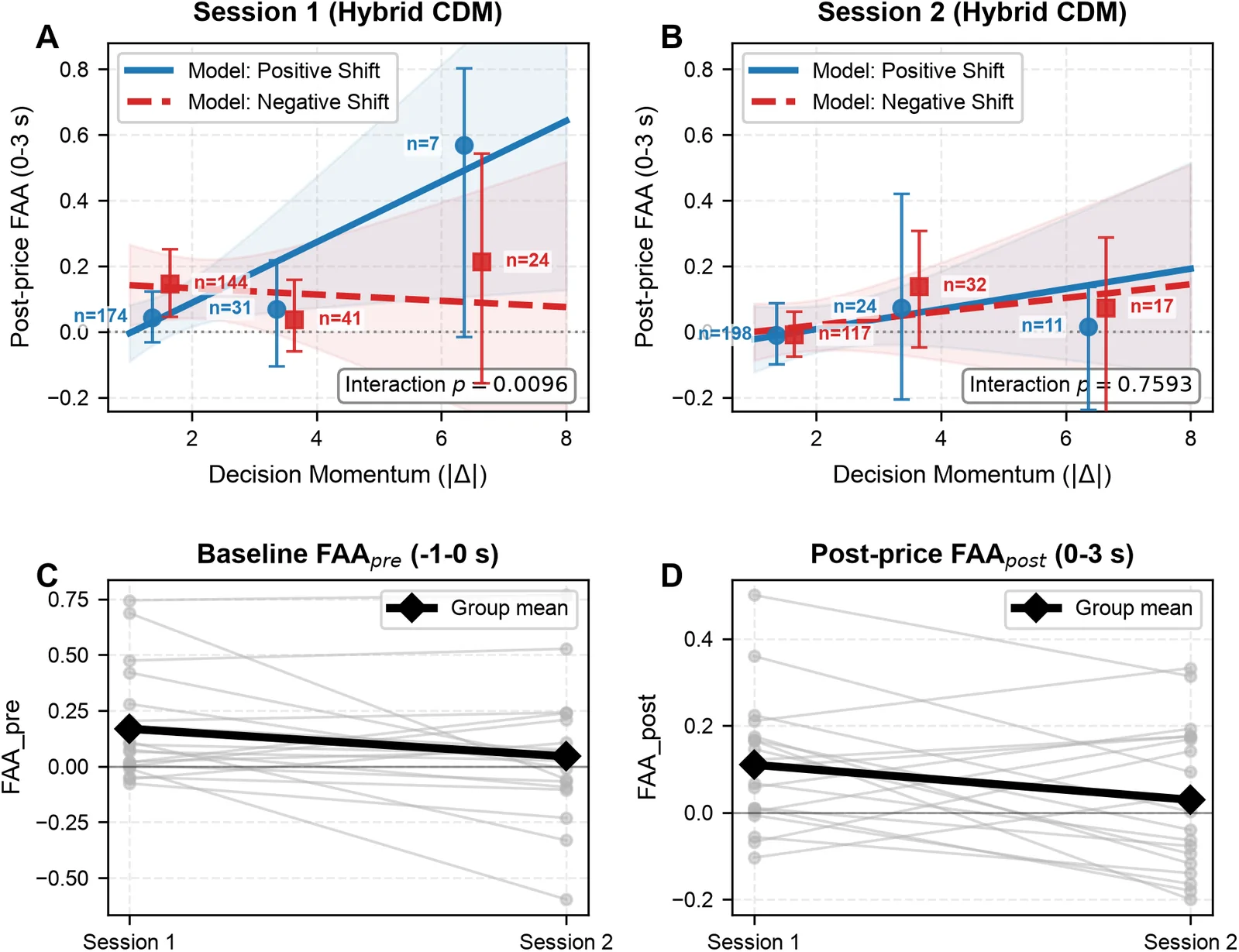
Rating-change magnitude and block-level FAA. **(A)** Session 1: FAA_post_ (0–3 s after price onset) as a function of rating-change magnitude (|Δ|), stratified by the direction of the change (Positive vs Negative shift). Solid/dashed lines show predictions from the session-stratified Core Decision–Momentum Model (Valence × magnitude + RatingProduct) fitted by ordinary least squares, with shaded bands giving 95% uncertainty from the participant-clustered robust covariance of that fit; the interaction *p*-values quoted in the panels come from the GEE fits of the same model reported in Table 4. Markers show binned mean FAA_post_ within Momentum ranges (1–2, 3–4, 5–8), with error bars indicating 95% cluster bootstrap intervals obtained by resampling participants with replacement. Labels “*n*” denote the number of *trials* contributing to each binned mean within that direction. Data are sparse in the highest bin—in Session 1, 7 Positive-shift trials from 2 participants have |Δ|≥5—so estimates in this range have sparse support and should not be read as effect-size estimates for large changes. **(B)** Session 2: same visualization as in (A). **(C)** Baseline FAA (FAA_pre_, −1 to 0 s) shown as participant-level means across retained trials within each session (each gray line = one participant; *N* = 19), with the bold line indicating the group mean. **(D)** Post-price FAA (FAA_post_, 0–3 s) shown as participant-level means across retained trials within each session (each gray line = one participant; *N* = 19), with the bold line indicating the group mean. Interaction *p*-values in (A–B) correspond to the Valence × Momentum term from the session-stratified robust models reported in Table 4.

**Where the Valence coefficient is evaluated:** Because the model contains Valence×|Δ|, the Valence coefficient is the direction contrast at |Δ|=0, a value that does not occur in the primary set. Re-centering makes its dependence explicit: pooled, the contrast is + 0.135 (*p* = 0.044) at |Δ|=0, + 0.084 (*p* = 0.035) at the observed minimum of 1, and + 0.033 (*p* = 0.16) at the sample mean of 2.00; in Session 1 the corresponding values are + 0.224 (*p* = 0.008), + 0.138 (*p* = 0.020), and + 0.050 (*p* = 0.26). The interaction is invariant to centering, as it must be. We therefore report the direction contrast only with its centering stated, and do not describe it as a main effect of shift direction.

### Between-block differences in baseline and post-price FAA

To determine whether the pooled session effect reflected a baseline shift or a task-specific modulation, we evaluated session differences in FAA_pre_ (−1 to 0 s), FAA_post_ (0–3 s), and their difference (*d*FAA). The session shift was significant for post-price FAA (GEE β=−0.0888, *p* = 0.0303) and ran in the same direction at baseline (FAA_pre_: GEE β=−0.0924, *p* = 0.0643), while the within-trial change did not differ by session (*d*FAA: GEE β=0.0045, *p* = 0.9399; Table 3). This convergent pattern—a same-direction session shift in both baseline and post-price FAA, together with an unchanged within-trial difference—is more consistent with a difference in the level from which both windows start than with a change in the within-trial response. To probe the nature of this tonic shift directly, we examined posterior alpha power—a standard EEG index of cortical arousal, here computed as *absolute* log alpha power averaged over centro-parietal sensors (P3/Pz/P4/C3/Cz/C4); we use the term “posterior” loosely for this centro-parietal pool, and the absolute (rather than relative) formulation is deliberate because absolute alpha power is the conventional arousal index and avoids the broadband denominator used for FAA—across sessions. Centro-parietal alpha power measured in the 0–3 s task window was higher in Session 2 (GEE β=0.1288, *SE* = 0.0405, 95% CI [0.049, 0.208], *p* = 0.0015; OLS cluster-robust β=0.1860, *p* = 0.0003; 15 of 19 participants showing the increase; Table 3). In a resting recording an increase of this kind is usually interpreted as reduced cortical arousal; because it was measured during the task rather than in a separate baseline, we treat reduced arousal as a possible interpretation rather than a demonstrated state. The within-trial change *d*FAA did not differ detectably between blocks (β=0.0045, 95% CI [−0.111,0.120], *p* = 0.94). Together the higher task-window alpha and the absence of a detectable change in *d*FAA are compatible with a state-related account of the between-block difference, but the design cannot separate that from fatigue, task adaptation, the different stimulus set used in Session 2, or recording-session factors, and establishes none of them as the cause. Posterior alpha and frontal asymmetry were only weakly correlated in the reported analysis rather than restatements of one another (within participants, r=−0.04), and the posterior-alpha session effect was unchanged when FAA was included as a covariate (GEE β=0.1267, *p* = 0.0017). The two measures therefore behave differently across blocks: one is a lateralized frontal difference, the other non-lateralized centro-parietal power. We do not label either with the psychological construct it is often taken to index, since neither construct was measured here.

**Table 3:** Robust tonic analyses of session effects. Session differences were evaluated for baseline FAA (FAA_pre_; −1 to 0 s), post-price FAA (FAA_post_; 0–3 s), task-related change ($dFAA={FAA}_{post}-{FAA}_{pre}$), and centro-parietal alpha power measured in the task window. Primary estimator: GEE (exchangeable). Sensitivity: OLS (cluster-robust).

Outcome	Term	Coef	SE	*p*	95% CI
**${FAA}_{pre}\sim Session$** (*N*_obs_ = 937, *N*_subj_ = 19)					
GEE	Intercept	0.1599	0.0520	0.0021	(0.0580, 0.2619)
GEE	Session 2^a^	−0.0924	0.0499	0.0643	(−0.1902, 0.0055)
OLS (cluster)	Intercept	0.1614	0.0520	0.0019	(0.0595, 0.2634)
OLS (cluster)	Session 2^a^	−0.0969	0.0509	0.0569	(−0.1966, 0.0029)
**${FAA}_{post}\sim Session$** (*N*_obs_ = 937, *N*_subj_ = 19)					
GEE	Intercept	0.1050	0.0324	0.0012	(0.0415, 0.1684)
GEE	Session 2^a^	−0.0888	0.0410	**0.0303**	(−0.1692, −0.0085)
OLS (cluster)	Intercept	0.0996	0.0320	0.0018	(0.0370, 0.1623)
OLS (cluster)	Session 2^a^	−0.0906	0.0428	**0.0341**	(−0.1745, −0.0068)
**dFAA~Session** (*N*_obs_ = 937, *N*_subj_ = 19)					
GEE	Intercept	−0.0564	0.0500	0.2601	(−0.1544, 0.0417)
GEE	Session 2^a^	0.0045	0.0590	0.9399	(−0.1112, 0.1201)
OLS (cluster)	Intercept	−0.0618	0.0524	0.2383	(−0.1646, 0.0409)
OLS (cluster)	Session 2^a^	0.0062	0.0601	0.9175	(−0.1116, 0.1241)
**Posterior Alpha ~Session**b (*N*_obs_ = 937, *N*_subj_ = 19)					
GEE	Intercept	−27.9791	0.1654	< 0.0001	(−28.3033, −27.6550)
GEE	Session 2^a^	0.1288	0.0405	**0.0015**	(0.0494, 0.2082)
OLS (cluster)	Intercept	−28.0100	0.1747	< 0.0001	(−28.3524, −27.6675)
OLS (cluster)	Session 2^a^	0.1860	0.0516	**0.0003**	(0.0848, 0.2872)

^a^Session is dummy-coded with Session 1 as the reference category.

^b^Posterior alpha = absolute log alpha power (8–13 Hz, Welch), averaged over the centro-parietal sensors P3/Pz/P4/C3/Cz/C4 in the 0–3 s task window; the measured quantity is absolute log alpha power in the task window; higher values are often interpreted as lower cortical arousal, but that interpretation was not tested here. Its intercept is on an absolute log-power scale and is therefore not directly comparable in magnitude to the relative-power FAA outcomes; the Session 2 contrast is the comparable quantity.

### Exploratory session-stratified models

The pooled test having been inconclusive, we fit the CDM separately within each session (Table 4). These stratified models were not pre-specified and are reported as exploratory throughout; they do not confer primary status on either session. In Session 1 the Valence × magnitude interaction was statistically significant under the principal specification (GEE: β=−0.0867, 95% CI [−0.152,−0.021], *p* = 0.0096; cluster-robust OLS: β=−0.1019, *p* = 0.0036), indicating that the magnitude–FAA slope differed by the direction of the rating change. In Session 2 no corresponding association was observed (interaction β=−0.0127, 95% CI [−0.094,0.068], *p* = 0.76; all terms *p* > 0.1). We describe this as a pattern observed in the first block of this dataset, not as a session effect that has been shown to replicate.

**Table 4:** Session-stratified robust models predicting FAA_post_ (CDM). Primary estimator: GEE (exchangeable). Sensitivity: OLS with cluster-robust standard errors.

Session	Estimator	Term	Coef	SE	*p*	95% CI
**Session 1** (*N*_obs_ = 421, *N*_subj_ = 19)						
S1	GEE	Intercept	−0.1503	0.1031	0.1448	(−0.3524, 0.0517)
S1	GEE	Valence (Negative)^a^	0.2243	0.0851	**0.0084**	(0.0575, 0.3911)
S1	GEE	Momentum (|Δ|)	0.0729	0.0380	0.0553	(−0.0017, 0.1474)
S1	GEE	Valence (Negative)×Momentum	−0.0867	0.0335	**0.0096**	(−0.1524, −0.0211)
S1	GEE	RatingProduct	0.0180	0.0139	0.1956	(−0.0093, 0.0452)
S1	OLS (cluster)	Intercept	−0.1743	0.1181	0.1398	(−0.4057, 0.0571)
S1	OLS (cluster)	Valence (Negative)^a^	0.2478	0.0904	**0.0061**	(0.0707, 0.4249)
S1	OLS (cluster)	Momentum (|Δ|)	0.0924	0.0411	**0.0245**	(0.0119, 0.1729)
S1	OLS (cluster)	Valence (Negative)×Momentum	−0.1019	0.0350	**0.0036**	(−0.1704, −0.0334)
S1	OLS (cluster)	RatingProduct	0.0151	0.0162	0.3515	(−0.0167, 0.0469)
**Session 2** (*N*_obs_ = 399, *N*_subj_ = 19)						
S2	GEE	Intercept	−0.1438	0.0964	0.1358	(−0.3327, 0.0451)
S2	GEE	Valence (Negative)^a^	0.0322	0.0858	0.7070	(−0.1359, 0.2003)
S2	GEE	Momentum (|Δ|)	0.0283	0.0277	0.3065	(−0.0259, 0.0825)
S2	GEE	Valence (Negative)×Momentum	−0.0127	0.0413	0.7593	(−0.0936, 0.0683)
S2	GEE	RatingProduct	0.0202	0.0123	0.1006	(−0.0039, 0.0443)
S2	OLS (cluster)	Intercept	−0.1594	0.0989	0.1072	(−0.3533, 0.0345)
S2	OLS (cluster)	Valence (Negative)^a^	0.0322	0.0882	0.7152	(−0.1407, 0.2050)
S2	OLS (cluster)	Momentum (|Δ|)	0.0307	0.0284	0.2800	(−0.0250, 0.0863)
S2	OLS (cluster)	Valence (Negative)×Momentum	−0.0100	0.0420	0.8127	(−0.0923, 0.0724)
S2	OLS (cluster)	RatingProduct	0.0207	0.0125	0.0983	(−0.0038, 0.0453)

^a^Valence is dummy-coded with Positive Shift as the reference category.

### Sensitivity analyses

The following checks establish what the reported estimate depends on. Each is given with coefficients and intervals rather than only whether *p* crosses 0.05, for the pooled model and for the exploratory Session 1 model (S5 Table).

(i) **Artifact-rejection threshold:** The 150 μV peak-to-peak criterion was the original specification; we refit after rerunning the entire preprocessing pipeline at 100 and 200 μV. Loosening the criterion to 200 μV retained more data (1,049 artifact-free trials; primary set *N* = 892, 19 participants) and did not weaken the estimates (pooled β=−0.0434, 95% CI [−0.091,0.004], *p* = 0.075; Session 1 β=−0.1004, 95% CI [−0.165,−0.036], *p* = 0.0022). Tightening it to 100 μV removed a large share of the data (737 artifact-free trials; primary set *N* = 510) and, critically, changed the analysis population: only 12 of 19 participants still met the screening rule, and the estimates moved toward zero (pooled β=+0.0093, *p* = 0.82; Session 1 β=−0.0477, 95% CI [−0.116,0.021], *p* = 0.17).

Because that comparison confounds retaining different trials with admitting different participants, we repeated it holding the participant set fixed at the 12 participants retained under every threshold. Within those 12, the Session 1 estimate was −0.0477 (*p* = 0.17) at 100 μV, −0.0522 (95% CI [−0.111,0.007], *p* = 0.084) at the published 150 μV, and −0.0627 (*p* = 0.044) at 200 μV, and the pooled estimate was near zero at all three. The estimate is therefore stable across the trial-level criterion but substantially smaller in this subgroup than in the full 19-participant set (−0.0867), which locates much of the difference between thresholds in *which participants* the screening rule admits rather than in which trials survive rejection. The association therefore persists, and is somewhat larger, under the more permissive threshold, while its size depends on which participants the screening rule admits.

(ii) **Leave-one-participant-out:** Refitting 19 times, each time omitting one participant, the Session 1 interaction ranged from −0.115 to −0.065 and remained below 0.05 in 18 of 19 folds; the least supportive fold (omitting S15) gave β=−0.0654, *p* = 0.056 under the primary GEE estimator (*p* = 0.036 under cluster-robust OLS). The pooled interaction ranged from −0.073 to −0.036 and reached *p* < 0.05 in only 2 of 19 folds, consistent with its inconclusive full-sample result. No single participant drives either estimate, but neither is the Session 1 result independent of the sample composition.
(iii) **Small-sample inference:** With 19 clusters, cluster-robust standard errors are optimistic. The bias-reduced (CR2) covariance with Satterthwaite degrees of freedom and the wild cluster bootstrap are both applied to the *ordinary-least-squares* fit, so they refer to the OLS coefficient rather than to the GEE estimate reported as primary, and their *p*-values should not be read as belonging to the same fitted quantity. For the pooled model the OLS interaction is β=−0.0562, *SE* = 0.0330, dof = 10.5, *p* = 0.118, with bootstrap *p* = 0.127 (GEE estimate β=−0.0510, *p* = 0.093). For Session 1 the OLS interaction is β=−0.1019, *SE* = 0.0386, dof = 9.0, *p* = 0.027, with bootstrap *p* = 0.020, against a nominal cluster-robust *p* = 0.0036 for the same coefficient and a GEE estimate of β=−0.0867, *p* = 0.0096. The corrections reduce the apparent strength of the Session 1 result and leave the pooled result unsupported. A random-intercept mixed model gave β=−0.080, *SE* = 0.042, *p* = 0.057; we note that this model is not itself free of small-sample assumptions and treat the spread across estimators as the honest range of uncertainty rather than as a demonstration that the effect is estimator-independent.
(iv) **Participant-inclusion rule:** The screening rule (at least three trials of each direction in each session) is a researcher degree of freedom, and we report it in both directions. Loosening it—readmitting the two excluded participants—left the Session 1 estimate essentially unchanged (β=−0.0818, 95% CI [−0.147,−0.017], *p* = 0.014, *N* = 427 trials from 20 participants; the 21st contributed no Session 1 trials that survived rejection) while the pooled estimate remained inconclusive (β=−0.0441, *p* = 0.15, *N* = 842 trials from 21 participants). Tightening it—counting only observed shift directions, so that the imputed direction of boundary trials no longer helps a participant qualify—excluded two further participants and attenuated the estimates (both sessions required: 17 participants, Session 1 β=−0.0625, 95% CI [−0.129,0.004], *p* = 0.066; Session 1 counts only: 18 participants, β=−0.0847, *p* = 0.012). The result is thus not produced by the exclusions, but its strength varies between *p* = 0.012 and *p* = 0.066 across defensible versions of a rule the data cannot settle.
(v) **Baseline-adjusted model:** Adding pre-price FAA as a covariate left the Session 1 interaction unchanged (β=−0.0859, 95% CI [−0.150,−0.022], *p* = 0.0082) and the pooled interaction inconclusive (β=−0.0481, *p* = 0.11), indicating that the association is not accounted for by measured pre-price variation in FAA.

Further checks reported previously were retained (S2 Table). Adding back the 117 boundary trials left the Session 1 interaction essentially unchanged (β=−0.090, *p* = 0.012; *N* = 474). Trials are crossed by participant and item (60 products); clustering by item preserved the effect (*p* = 0.009), whereas a crossed mixed-effects model with random intercepts for participant and item reduced it to β=−0.075, *p* = 0.075, so item dependence is not a technicality here. Adding a within-category price-deviation covariate left the interaction intact (β=−0.093, *p* = 0.005); price deviation was not itself associated with FAA (Session 1: *p* = 0.19) and correlated only weakly with |Δ| (*r* = 0.18).

(vi) **FAA operationalization:** The primary feature is relative alpha power at F3/F4 on average-referenced data with a fixed 8–13 Hz band. We refit the Session 1 model under five alternatives (S4 Table). Two alternative frontal pairs gave estimates that do not reproduce the primary one but are imprecise: F7/F8 β=+0.023, 95% CI [−0.085,0.131], *p* = 0.67; AF3/AF4 β=−0.066, 95% CI [−0.180,0.048], *p* = 0.26. Removing the broadband denominator (absolute log alpha) gave β=−0.034, 95% CI [−0.080,0.012], *p* = 0.15 (*N* = 474, the retained set); a surface-Laplacian (current-source-density) transform gave β=+0.005, 95% CI [−0.047,0.058], *p* = 0.84; and an individualized alpha band gave β=−0.032, 95% CI [−0.124,0.060], *p* = 0.49. For comparison on the same trials, the primary feature gave β=−0.090, 95% CI [−0.159,−0.020], *p* = 0.012.

Each row is reported as its own estimate with its own uncertainty, and none of them is evaluated against the F3/F4 estimate as though that were a known value: the alternative pairs measure a different scalp contrast, and the surface-Laplacian and individualized-band features are on different scales, so the coefficients are not on a common footing. The current-source-density and individualized-alpha features are also on different scales from relative-power FAA, so their coefficients are not directly comparable in magnitude. It is plausible that a 15-electrode montage limits the Laplacian estimate and that narrowing the band reduces the number of spectral bins, but we did not test either explanation and do not assert it. Statistical support was obtained under the primary average-referenced, fixed-band F3/F4 specification but was not consistently retained across alternative operationalizations. These results leave the robustness and generalizability of the association uncertain.

We also note a property of the measure itself. Because the relative-power form can be written as $FAA=\log(P_{\alpha ,F4}/P_{\alpha ,F3})-\log(P_{total,F4}/P_{total,F3})$, it is an alpha asymmetry adjusted by a broadband asymmetry, so broadband differences between the two sites can influence it; the absolute-alpha row above is the corresponding check, and it did not reproduce the effect.

**Permutation test:** Shift direction was permuted within each participant, 5,000 times, and the Session 1 least-squares interaction recomputed; the null is that direction carries no information about FAA beyond each participant’s own class balance, and exchangeability is assumed within participant and not across. An unconstrained shuffle can generate trials the 1–9 scale makes impossible—in Session 1, 81 of 421 trials (19.2%) cannot carry the opposite label, and the two predictors are coupled in the data (mean |Δ|=1.77 on Positive-shift versus 2.25 on Negative-shift trials)—so the reported test permutes only relabelings the scale admits, giving a two-sided empirical *p* = 0.006 (the unconstrained version gives *p* = 0.007). Restricting the permutation to feasible combinations makes the null better defined but does not by itself establish that the exchangeability assumption holds, and we do not treat this test as decisive on its own.

### Is the association specific to the post-price window?

Both predictors are derived from the post-price rating, which the participant has not yet given during the pre-price fixation. An interaction in the −1 to 0 s window therefore cannot be a response to the price, which makes that window a check on what the post-price estimate can be attributed to. We report it here because it bears directly on the interpretation of every result above (S6 Table).

**The same model in each window:** Fitting the CDM with pre-price FAA as the outcome gave, pooled, β=−0.1081, 95% CI [−0.168,−0.048], *p* = 0.0004—larger than the post-price estimate in the same data (β=−0.0510, 95% CI [−0.111,0.009], *p* = 0.093). In Session 2 the same reversal was clearer (pre-price β=−0.1258, 95% CI [−0.203,−0.048], *p* = 0.0015; post-price β=−0.0127, *p* = 0.76). In Session 1 the two windows were of similar size in the same direction (pre-price β=−0.0791, 95% CI [−0.168,0.010], *p* = 0.082; post-price β=−0.0867, 95% CI [−0.152,−0.021], *p* = 0.0096). Under the CR2 small-sample correction, applied to the corresponding least-squares fits, the pre-price estimate remained significant pooled (*p* = 0.029) and in Session 2 (*p* = 0.028) but not in Session 1 (*p* = 0.138, against *p* = 0.027 for the post-price window).

**The direct test of a window difference:** A difference in significance between two windows is not a test that the windows differ, so we tested it directly using the within-trial change $dFAA={FAA}_{post}-{FAA}_{pre}$, whose interaction term estimates that difference directly. Because each model is fitted separately by GEE, this coefficient is close to, but not identical with, the arithmetic difference of the two window-specific coefficients (−0.0081 against −0.0076 in Session 1). The estimated difference was −0.0081 in Session 1 (95% CI [−0.125,0.108], *p* = 0.89) and + 0.0574 pooled (95% CI [−0.026,0.141], *p* = 0.18); in Session 2 it was + 0.1089 (95% CI [0.017, 0.201], *p* = 0.021), the block in which the pre-price window carried the association and the post-price window did not. *For Session 1, therefore, the direct comparison did not establish a difference between the windows*: the estimated difference is close to zero with an interval spanning both directions, so neither equality nor difference is supported.

**Screening the baseline window:** The pre-price window was never screened for amplitude artifacts by the published pipeline, which determined rejection from the 0–3 s task window only. Applying the same 150 μV criterion to the pre-price window removed 38 of 820 trials (4.6%) and did not attenuate the association (pooled pre-price β=−0.1158, 95% CI [−0.176,−0.056], *p* = 0.0002; Session 1 β=−0.0961, 95% CI [−0.187,−0.006], *p* = 0.038). Amplitude screening of that window therefore does not remove the effect; it does not follow that ocular or muscular contamination has been excluded, and we make no such claim. Likewise, the trial-level correlation between the two windows is low (*r* = 0.06), but we did not quantify how much signal the filtering could displace across the window boundary, so temporal smearing remains unquantified here rather than ruled out.

**What this does and does not license:** Adjusting the post-price model for pre-price FAA left the Session 1 estimate unchanged (β=−0.0859, 95% CI [−0.150,−0.022], *p* = 0.0082), so the association is not accounted for by measured pre-price variation in FAA. At the same time, an interaction of comparable or larger size is present before price onset in the pooled and Session 2 data, and in Session 1 the direct comparison did not establish a difference between the windows. Attribution of the association specifically to price-related evaluative processing is therefore not supported by these data. Several accounts remain open and this design cannot separate them: a transient state fluctuation preceding large revisions; activity related to producing the ratings themselves, which bracket the price display on both sides; or the number of window-by-stratum comparisons made. Distinguishing them requires a baseline recorded away from the rating—an earlier resting block, or a long jittered gap between the pre-price response and price onset—rather than the second immediately preceding the event.

### Exploratory three-way interaction with session

Building upon the session-stratified observations (which suggested the momentum-FAA relationship was specific to Session 1), we conducted an exploratory test for session-to-session change by fitting a 3-way interaction Generalized Estimating Equation (GEE) model: FAA~Valence×Momentum×Session+RatingProduct. Because this analysis was prompted by the session-stratified results rather than pre-specified, it is reported as exploratory.

The three-way term was β=0.0969, *SE* = 0.0416, 95% CI [0.0154, 0.1783], *p* = 0.0198, indicating that the estimated slope difference was not the same in the two blocks. Because the blocks differ in stimulus set as well as in order, this term describes variation between session/stimulus-set combinations and cannot separate habituation, fatigue, or state change from properties of the two product sets. The test was prompted by the stratified results rather than pre-specified, rests on 19 participant clusters, and is one of several comparisons reported here; it is hypothesis-generating and we do not treat it as establishing a session-dependent mechanism.

### Single-trial classification of change direction from FAA

To complement the population-level inference, we asked whether FAA_post_ alone could classify the direction of a trial’s rating change out of sample, using logistic regression with leave-one-participant-out cross-validation (participants held out entirely, preventing leakage) and evaluating performance across increasingly stringent magnitude thresholds (|Δ|≥1,2,3,4,5).

**Principal classification (observed shifts only):** A 9→9 trial is not an observed Positive shift and a 1→1 trial is not an observed Negative shift, so the principal classification uses only trials with an observed direction (*N* = 820; 445 Positive, 375 Negative). Performance was above the within-participant permutation null (accuracy 54.0%, balanced accuracy 53.9%, ROC AUC 0.538, one-sided permutation *p* = 0.027), while the participant bootstrap interval included chance (ROC AUC 95% CI [0.476, 0.564]). The permutation analysis evaluates performance relative to a within-participant label-shuffling null, whereas the participant bootstrap estimates uncertainty under resampling of participants. Taken together they indicate *modest performance with uncertain generalizability*: the observed performance advantage was small, and it is not established that it would transfer to new participants.

**Across magnitude thresholds:** The same analysis was repeated at |Δ|≥1 through |Δ|≥5 (S1 Table). Performance did not improve as the threshold rose: ROC AUC was 0.538, 0.419, 0.392, 0.379 and 0.317, with permutation p≥0.85 above the baseline threshold and bootstrap intervals that widen sharply (at |Δ|≥5, [0.136, 0.688]). The higher thresholds are also unstable: contributing participants fall from 19 to 15, and held-out participants represented by only one class rise to 11 of 17 at |Δ|≥4 and 11 of 15 at |Δ|≥5, so values below chance there indicate no reliable signal rather than systematic misclassification.

**Directional recoding as a sensitivity analysis:** The submitted version instead assigned the 117 boundary trials a direction and a magnitude of one. Because those trials carry |Δ|=1, the two codings can differ only at the baseline threshold, and they do: *N* = 937, ROC AUC 0.528, permutation *p* = 0.093, bootstrap [0.469, 0.555], against 0.538 and *p* = 0.027 under the principal coding. From |Δ|≥2 upward the two series are identical. Both support the same reading; we report the observed-shift version as principal because the recoded direction is an assumption rather than a measurement (S1 Table gives both).

These results delimit what the population-level analyses can be taken to show. An association estimated across participants does not imply that the same feature predicts an individual trial in a new participant, and here it did not.

## Discussion

### What the primary and exploratory analyses support

The primary pooled analysis provided uncertain evidence that the association between rating-change magnitude and FAA_post_ differed by the direction of the change (β=−0.051, 95% CI [−0.111,0.009], *p* = 0.093; for the corresponding least-squares estimate, CR2 *p* = 0.118 and bootstrap *p* = 0.127). An exploratory session-stratified analysis identified an interaction in Session 1 under the average-referenced, fixed-band F3/F4 specification (β=−0.087, 95% CI [−0.152,−0.021], *p* = 0.010; for the corresponding least-squares estimate, CR2 *p* = 0.027, bootstrap *p* = 0.020 and constrained permutation *p* = 0.006). Statistical support varied across modeling choices and EEG operationalizations: a crossed participant-and-item model gave *p* = 0.075, an absolute-alpha formulation *p* = 0.15, and alternative electrode pairs, a surface-Laplacian transform and an individualized alpha band did not reproduce it. These findings motivate further investigation but do not establish a general relationship between frontal alpha asymmetry and the intensity of consumer decisions.

Two further constraints bound the interpretation. First, the exploratory estimate is a difference between two slopes, neither of which is individually distinguishable from zero (Session 1 Positive-shift slope + 0.073, 95% CI [−0.002,0.147]; Negative-shift slope −0.014, 95% CI [−0.084,0.057]; Table 2), and the predicted contrast between a one-point and a five-point change has an interval that includes zero (+ 0.29, 95% CI [−0.01,0.59]) and rests on 7 trials from 2 participants at its upper end. Second, and most consequentially, an interaction of comparable or larger size appears in the window *before* the price is displayed in the pooled and Session 2 data, and in Session 1 the pre- and post-price windows cannot be shown to differ (within-trial change β=−0.008, *p* = 0.89). Adjusting for pre-price FAA leaves the Session 1 estimate intact (β=−0.086, *p* = 0.008), so the association is not explained by measured baseline variation; but attributing it specifically to price-related evaluative processing is not supported by these data.

We therefore read the result as follows: *an exploratory association between the direction and magnitude of an instructed rating change and post-price frontal alpha asymmetry was observed in Session 1 under the principal specification, the baseline findings complicate attributing that association specifically to price evaluation, and the finding is hypothesis-generating rather than evidence of an established neural mechanism of price evaluation.* What the analyses license is a statement about the statistical results obtained under the reported specification, not a claim that a phenomenon has been demonstrated.

### Positioning relative to prior FAA theory and methodological work

FAA has a long history as a theory-driven EEG marker commonly interpreted within approach–withdrawal and affective-style frameworks, where frontal asymmetry patterns are linked to motivational and regulatory tendencies [4,6]. In this context, FAA is typically operationalized using alpha-band activity over homologous frontal sites, with alpha power inversely related to cortical activation. Reviews have emphasized that frontal EEG asymmetry can reflect both relatively stable individual differences and state-dependent fluctuations, and that careful operational choices (e.g., montage/reference, preprocessing, and quality control) are critical for interpretable inference [5,6].

Consistent with this broader literature, the two blocks of the present design differed in FAA level, motivating an explicit baseline check. Classic psychometric work shows that resting anterior EEG asymmetry can exhibit meaningful temporal stability, yet it also varies with context and measurement conditions [5,11]. Therefore, rather than treating across-session shifts as purely task-evoked differences, it is methodologically prudent to evaluate whether session effects reflect baseline/state changes that carry into task windows. This logic directly informed our decision to examine the pre-price window directly rather than assume that a between-block difference reflects a change in the within-trial response (Table 3 and Fig 3C–3D).

### What could the association mean? Three accounts the data cannot separate

FAA was quantified as $\ln(Rel\alpha_{F4})-\ln(Rel\alpha_{F3})$ over the 0–3 s window following price onset. It is a lateralized scalp measure of alpha-band power at two frontal electrodes; we refer to it as frontal alpha asymmetry recorded at F3/F4 and do not equate it with localized prefrontal activation, with approach motivation, or with decision intensity, none of which were measured here.

At least three accounts are compatible with the present pattern, and this design cannot distinguish them.

*Motivational direction (approach–withdrawal):* The framework within which FAA is usually interpreted holds that relatively greater left-frontal alpha suppression accompanies approach-related states [4–6]. It predicts direction-dependent slopes of the kind observed in Session 1, and it is the account the design was built around. Against it, approach motivation was not independently measured, so this reading is imported from prior literature rather than established here; and the interaction is not confined to the window in which a price-driven motivational response would have to occur.
*Effort, conflict, or evaluative revision:* A large revision of a rating implies a larger discrepancy between a prior evaluation and the newly available information, which recruits cognitive control rather than motivational direction specifically. This account predicts that the clearest signature should be non-lateralized—mid-frontal theta, response-locked control signals, or a slower evaluative component—rather than a lateralized alpha asymmetry, and it would also predict activity spanning the whole revision process rather than an event-locked response to the price.
*Attention and engagement:* Alpha power indexes cortical engagement, and lateralized alpha differences can arise from asymmetric attentional allocation to the display rather than from evaluation as such. This account would also accommodate an association that appears both before and after price onset; like the others, it was not measured here, and we do not rank the three.

We introduced posterior alpha earlier as an arousal index for the between-block difference, and it should not be extended beyond that. It was computed in the 0–3 s task window rather than in a separate resting baseline, so it is not a direct measure of tonic arousal; it was uncorrelated with FAA within participants (r=−0.04); and the per-participant change in posterior alpha did not predict the per-participant attenuation of the momentum-related estimate. Arousal is therefore a hypothesis about the between-block difference, not an explanation of the FAA–magnitude association.

Separating these accounts requires measurements this study did not make. Independent indices of motivational disposition (for example, behavioral approach/inhibition scales), of effort and conflict (mid-frontal theta, response times), and of arousal (pupil diameter or electrodermal activity recorded outside the rating window) would each be diagnostic; so would a baseline recorded away from the participant’s own rating response, which the present pre-price window is not.

### The between-block difference and its interpretation

FAA_post_ was lower in the second block than in the first (β=−0.0888, 95% CI [−0.169,−0.008], *p* = 0.030), and the difference ran in the same direction before price onset (FAA_pre_: β=−0.0924, 95% CI [−0.190,0.006], *p* = 0.064), while the within-trial change did not differ detectably between blocks (*d*FAA: β=0.0045, 95% CI [−0.111,0.120], *p* = 0.94; Table 3). The last of these is a wide interval containing the published post-price difference, so it indicates that no clear difference was detected, not that the within-trial change was equivalent across blocks. Taken together the three estimates are more consistent with a difference in the level from which both windows start than with a change in the within-trial response, but they do not establish that.

Posterior alpha power over centro-parietal sensors was higher in the second block (β=0.1288, 95% CI [0.049, 0.208], *p* = 0.0015), which in a resting recording would be read as reduced cortical arousal. Here it was computed in the 0–3 s task window rather than in a separate baseline, so it is an index of task-window alpha rather than of tonic arousal as such; and the per-participant increase did not predict the per-participant attenuation of the momentum-related estimate. We therefore treat reduced arousal or disengagement as one hypothesis about the between-block difference rather than as its demonstrated cause, and we do not use it as a reason to give Session 1 primary status.

The design also limits what any between-block estimate can mean. Block order and stimulus set were fully confounded: all participants viewed set A first and set B second, and their median displayed prices were THB 618 and THB 339, respectively. Time, fatigue, habituation, and the products themselves are therefore not separable here. Psychometric and review work on frontal asymmetry indicates that the measure contains both trait-like and state-dependent components, which is why we assessed baseline windows directly rather than assuming them away [5,6,11].

### Role of product liking

Higher product liking was associated with higher FAA_post_ in the pooled model, but the interval is compatible with no association (β=0.0178, 95% CI [−0.0009,0.0365], *p* = 0.062). Prior work reports that EEG power over frontal and centro-parietal sites differentiates liking from disliking decisions [13]; the present estimate is consistent with that literature in sign but too imprecise to add support to it, and we retain the term as a covariate rather than as a finding.

### Methodological implications

Three points follow from how the analyses behaved rather than from the size of any estimate. First, the pre-price window is worth analyzing as an outcome in its own right in designs of this kind. Because the predictors here are derived from a response the participant has not yet made, that window functions as a check on temporal attribution, and it changed what we could claim; studies that report only the post-stimulus window cannot perform that check. Second, in repeated-block designs the between-block level of FAA should be examined directly, since both the baseline and the task window shifted here while the within-trial change did not. Third, population-level association and single-trial prediction are separate questions with separate answers: the association estimated across participants did not translate into reliable out-of-participant classification, and neither result substitutes for the other.

We also note what this study cannot support. A single asymmetry index evaluated on 19 participants, with the sensitivity profile reported above, does not constitute a validated marker of consumer decision processes, and classification performance with uncertain generalizability does not license describing FAA as a population-level biomarker. The contribution here is a transparent test of a theory-driven feature against an explicitly defined behavioral quantity, together with the boundaries of what that test can establish.

### Limitations and future directions

The limitations below are ordered by how much they restrict interpretation.

**The association is not shown to be time-locked to price onset:** An interaction of comparable or larger size is present in the pre-price window in the pooled and Session 2 data, and in Session 1 the two windows cannot be shown to differ. This is the limitation that most directly constrains the study’s original question, and it is why we do not describe the result as a price-evoked response. A design with a baseline recorded away from the participant’s own rating—an earlier resting block, or a long jittered interval between the pre-price response and price onset—would be required to separate a response to the price from a state preceding a large revision, or from processes related to producing the ratings that bracket the price display.

**The rating change was instructed, not spontaneous:** Participants were told that the post-price rating had to differ from the pre-price rating except at the scale limits, so a non-zero change was expected by design and compliance was complete (no non-boundary zero-change trials in 1,260 recorded trials). Nothing here shows that price exposure by itself produces a rating change, and part of the observed magnitude may reflect task compliance and any demand characteristic arising from a participant’s inference that the study concerned price. In addition, the bounded scale means a participant who gave an extreme pre-price rating could only move inward, so direction is partly a mechanical consequence of the first rating. A design permitting Δ=0 would remove the forced-change requirement, though it would not remove the floor and ceiling constraints of a bounded scale.

**Rating-change magnitude is not a validated construct:** No external criterion—willingness to pay, purchase intention, decision confidence, or response time—was collected, and no purchase was measured, so the quantity we model is the size of an instructed rating revision and nothing more. Its relationship to purchase behavior is untested, and the label “Decision Momentum” should be read as a provisional operational term rather than as a claim about decisiveness or persistence. Future work should collect |Δ| alongside willingness to pay (for example, an incentive-compatible auction), purchase intention, decision confidence, response time, and, where possible, actual choice, and test whether they converge; a condition without the change instruction would establish how much of the magnitude is spontaneous.

**Statistical support depends on choices the data cannot settle:** The primary pooled test was inconclusive; the exploratory Session 1 estimate weakened under a crossed participant-and-item model (*p* = 0.075), varied between *p* = 0.012 and *p* = 0.066 across defensible participant-screening rules, and was smaller among the 12 participants retained under every artifact threshold. With 19 clusters the effective degrees of freedom for the Session 1 estimate are 9.0 rather than the nominal 18. No a priori power analysis was conducted; the sample size was chosen to evaluate feasibility and the analytic workflow. All inferential results should be treated as preliminary and in need of an adequately powered, pre-registered replication.

**Session is confounded with stimulus set:** The fixed order (set A first, set B second) means no between-block estimate has a unique temporal interpretation, and the two sets differed in median price by a factor of 1.8. Counterbalancing stimulus sets across blocks, or modeling stimulus-set properties directly, is required.

**The measure is operationalization-dependent:** Statistical support was obtained under the average-referenced, fixed-band relative-alpha F3/F4 specification but was not consistently retained under alternative electrode pairs, absolute alpha, a surface-Laplacian transform or an individualized band. Each of those estimates is individually imprecise and several are on different scales (S4 Table), so they are not read as tests of the F3/F4 estimate, and the robustness and generalizability of the association remain uncertain. Denser montages would permit more reliable current-source-density estimation and linked-mastoid referencing.

**Boundary trials carry an assumption:** On 9→9 and 1→1 trials no change was observed, yet the original coding assigned a direction and a magnitude of one point. The primary models exclude all 117 such trials and the principal classification now uses observed shifts only; the directional recoding is reported as a sensitivity analysis. A censored or ordinal model treating these as bounded observations would be the principled alternative and was not implemented.

**Sample and generalizability:** The sample was young (18–27), university-recruited, unbalanced by sex (12 male, 7 female), and selected against an income ceiling that was recorded only as an eligibility check, so income variation could not be adjusted for even though purchasing power plausibly moderates how far a price moves an evaluation. Eligibility rested on self-report without documentary verification. The within-category price-deviation covariate captured only price extremity, not each participant’s subjective price expectation. Findings should not be extended to older or higher-income consumers, or to purchase decisions with real financial consequences.

## Conclusion

This pilot study tested whether frontal alpha asymmetry recorded at F3/F4 while a price was displayed varies with the direction and magnitude of an instructed satisfaction-rating change. The primary pooled test was inconclusive (β=−0.051, 95% CI [−0.111,0.009], *p* = 0.093): its interval includes both zero and effects of the size estimated in the first block (β=−0.087, 95% CI [−0.152,−0.021]), so the study neither established the association nor ruled it out.

An exploratory session-stratified analysis identified an interaction in the first block under the average-referenced, fixed-band F3/F4 specification. Its support varied across modeling choices and EEG operationalizations, and the same model applied to the window before the price appeared produced an interaction of comparable or larger size in the pooled and second-block data, with the direct comparison failing to establish a difference between windows in Session 1. We therefore report the association as hypothesis-generating and do not attribute it specifically to price-related evaluative processing. At the single-trial level, classification of change direction from FAA alone did not demonstrate reliable out-of-participant predictive performance.

What the study contributes is a transparent test of a theory-driven EEG feature against an explicitly defined behavioral quantity, together with the checks that bound it: a signed-change parameterization that makes the construct’s structure explicit, a sensitivity package spanning preprocessing thresholds, participant screening and small-sample inference, and a temporal-specificity check that constrains what the estimate can be attributed to. Answering the original question will require a pre-registered, adequately powered, counterbalanced design with a baseline recorded away from the rating response, an unconstrained rating condition, and criterion measures against which rating-change magnitude can be validated.

## Supporting information

S1 FigPooled hybrid visualization of the Core Decision–Momentum Model (CDM).Hybrid CDM plot for the pooled dataset showing FAA_post_ (0–3 s) as a function of rating-change magnitude (|Δ|), stratified by the direction of the change (Positive vs Negative shift). Lines indicate model-implied trends from the pooled CDM; shaded bands indicate 95% uncertainty for visualization. Markers show binned trial means with uncertainty intervals, and labels indicate the number of trials per bin. The interaction *p*-value corresponds to the Valence × Momentum term from the pooled GEE analysis reported in Table 1.(TIF)

S1 TableSingle-trial classification of change direction from FAA_post_ across rating-change magnitude thresholds.Logistic regression with leave-one-participant-out cross-validation, evaluated on trials at or above each threshold, under both codings of the scale-boundary trials: the principal coding, which uses observed shifts only, and the sensitivity coding, which assigns those trials a direction and a magnitude of one. For each row: trial count, class counts, accuracy, balanced accuracy, ROC AUC, the one-sided *p*-value from a within-participant label permutation (1,000 permutations; the proportion of permuted ROC AUCs at or above the observed value, so below-chance performance yields a value near 1), the participant bootstrap 95% confidence interval for ROC AUC (2,000 resamples of participants with replacement, all copies of a resampled participant held out together, classifier refitted within each resample), the number of contributing participants, and the number of held-out participants represented by only one class. The two codings can differ only at the baseline threshold, because boundary trials carry |Δ|=1.(CSV)

S2 TableAdditional robustness checks for the exploratory Session 1 direction × magnitude interaction.Each row reports the interaction coefficient (β), its *p*-value, and the number of trials (*N*_obs_) under an alternative specification, computed on the primary momentum set (Session 1, *N* = 421).(CSV)

S3 TableSigned change score: nested model comparison.Four specifications fitted to the primary momentum set (*N*_obs_ = 820): a linear term in the signed change Δ; a two-slope model allowing the slope to differ by direction; the Core Decision–Momentum Model, which adds a discontinuity at Δ=0; and signed change entered as a factor. For each, the number of parameters, AIC, BIC and adjusted *R*^2^ are reported. These information criteria come from ordinary least squares and do not account for the repeated-measures structure, so they are descriptive only. The two steps that differ by a single parameter are therefore also tested on the coefficient itself, by GEE with cluster-robust covariance and by a wild cluster bootstrap on the least-squares fit, and those results are given in the same table; the step that adds twelve parameters at once has no dependence-aware test here and is left to the information criteria.(CSV)

S4 TableFAA operationalization sensitivity.The Session 1 direction × magnitude interaction under six operationalizations of frontal alpha asymmetry: the primary relative-power F3/F4 measure on average-referenced data with a fixed 8–13 Hz band; the F7/F8 and AF3/AF4 pairs; absolute log alpha power without the broadband denominator; a surface-Laplacian (current-source-density) transform; and an individualized alpha band. Each row reports β, *SE*, 95% CI, *p* and the number of trials. The last three rows are computed on the retained set including scale-boundary trials, and the primary measure is repeated on those same trials for comparison. Coefficients of the current-source-density and individualized-band features are on different scales from the relative-power measure and are not directly comparable in magnitude.(CSV)

S5 TableSensitivity package.Coefficients, standard errors, 95% confidence intervals, trial counts and participant counts for the pooled and Session 1 direction × magnitude interaction under: (i) artifact-rejection thresholds of 100, 150 (original) and 200 μV, each rerun through the complete preprocessing pipeline, and repeated with the participant set held fixed at those retained under every threshold; (ii) leave-one-participant-out refitting; (iii) bias-reduced (CR2) covariance with Satterthwaite degrees of freedom and a wild cluster bootstrap; (iv) loosened and tightened participant-screening rules; and (v) adjustment for pre-price FAA.(CSV)

S6 TableTemporal specificity.The same model fitted with three outcomes—post-price FAA (0–3 s), pre-price FAA (−1 to 0 s), and the within-trial change—for the pooled data and each session, with β, 95% CI and *p*, together with CR2-corrected *p*-values, the post-price model adjusted for pre-price FAA, and a repetition of the pre-price analyses after applying the 150 μV criterion to the pre-price window.(CSV)

S1 Appendix Eligibility screening form. The items used at recruitment to assess the inclusion and exclusion criteria, as administered by the research team.
(PDF)

## References

[1] Ariely D, Berns GS. Neuromarketing: The hope and hype of neuroimaging in business. Nat Rev Neurosci. 2010;11(4):284–92. doi: 10.1038/nrn2795 20197790 PMC2875927

[2] Lim WM. Demystifying neuromarketing. J Bus Res. 2018;91:205–20. doi: 10.1016/j.jbusres.2018.05.036

[3] Byrne A, Bonfiglio E, Rigby C, Edelstyn N. A systematic review of the prediction of consumer preference using EEG measures and machine-learning in neuromarketing research. Brain Inform. 2022;9(1):27. doi: 10.1186/s40708-022-00175-3 36376735 PMC9663791

[4] Davidson RJ. Affective style and affective disorders: perspectives from affective neuroscience. Cogn Emot. 1998;12(3):307–30. doi: 10.1080/026999398379628

[5] Coan JA, Allen JJB. Frontal EEG asymmetry as a moderator and mediator of emotion. Biol Psychol. 2004;67(1–2):7–49. doi: 10.1016/j.biopsycho.2004.03.002 15130524

[6] Allen JJB, Keune PM, Schönenberg M, Nusslock R. Frontal EEG alpha asymmetry and emotion: From neural underpinnings and methodological considerations to psychopathology and social cognition. Psychophysiology. 2018;55(1):e13028. doi: 10.1111/psyp.13028 29243266

[7] Panda D, Chakladar DD, Rana S, Parayitam S. An EEG ‐based neuro‐recommendation system for improving consumer purchase experience. J Consum Behav. 2024;23(1):61–75. doi: 10.1002/cb.2142

[8] Ouzir M, Chakir Lamrani H, Bradley RL, El Moudden I. Neuromarketing and decision-making: Classification of consumer preferences based on changes analysis in the EEG signal of brain regions. Biomed Signal Process Control. 2024;87:105469. doi: 10.1016/j.bspc.2023.105469

[9] Kalaganis FP, Georgiadis K, Oikonomou VP, Laskaris NA, Nikolopoulos S, Kompatsiaris I. A hybrid neuromarketing approach exploiting EEG graph signal processing and gaze dynamic patterning. Brain Inform. 2025;12(1):23. doi: 10.1186/s40708-025-00272-z 40987926 PMC12457268

[10] Horr NK, Han K, Mousavi B, Tang R. Neural signature of buying decisions in real-world online shopping scenarios – An exploratory electroencephalography study series. Front Hum Neurosci. 2022;15:797064. doi: 10.3389/fnhum.2021.797064 PMC8882609 35237138

[11] Tomarken AJ, Davidson RJ, Wheeler RE, Kinney L. Psychometric properties of resting anterior EEG asymmetry: Temporal stability and internal consistency. Psychophysiology. 1992;29(5):576–92. doi: 10.1111/j.1469-8986.1992.tb02034.x 1410187

[12] Schöne B, Schomberg J, Gruber T, Quirin M. Event-related frontal alpha asymmetries: Electrophysiological correlates of approach motivation. Exp Brain Res. 2016;234(2):559–67. doi: 10.1007/s00221-015-4483-6 26537961

[13] Golnar-Nik P, Farashi S, Safari M-S. The application of EEG power for the prediction and interpretation of consumer decision-making: A neuromarketing study. Physiol Behav. 2019;207:90–8. doi: 10.1016/j.physbeh.2019.04.025 31047949

[14] Ravaja N, Somervuori O, Salminen M. Predicting purchase decision: The role of hemispheric asymmetry over the frontal cortex. J Neurosci Psychol Econ. 2013;6(1):1–13. doi: 10.1037/a0029949

[15] Ramsøy TZ, Skov M, Christensen MK, Stahlhut C. Frontal brain asymmetry and willingness to pay. Front Neurosci. 2018;12:138. doi: 10.3389/fnins.2018.00138 29662432 PMC5890093

[16] Pernet C, Garrido MI, Gramfort A, Maurits N, Michel CM, Pang E, et al. Issues and recommendations from the OHBM COBIDAS MEEG committee for reproducible EEG and MEG research. Nat Neurosci. 2020;23(12):1473–83. doi: 10.1038/s41593-020-00709-0 32958924

[17] Keil A, Debener S, Gratton G, Junghöfer M, Kappenman ES, Luck SJ, et al. Committee report: Publication guidelines and recommendations for studies using electroencephalography and magnetoencephalography. Psychophysiology. 2014;51(1):1–21. doi: 10.1111/psyp.12147 24147581

[18] Harrewijn A, Buzzell GA, Debnath R, Leibenluft E, Pine DS, Fox NA. Frontal alpha asymmetry moderates the relations between behavioral inhibition and social-effect ERN. Biol Psychol. 2019;141:10–6. doi: 10.1016/j.biopsycho.2018.12.014 30599209 PMC6471600

[19] Gramfort A, Luessi M, Larson E, Engemann DA, Strohmeier D, Brodbeck C, et al. MEG and EEG data analysis with MNE-Python. Front Neurosci. 2013;7:267. doi: 10.3389/fnins.2013.00267 24431986 PMC3872725

[20] Gramfort A, Luessi M, Larson E, Engemann DA, Strohmeier D, Brodbeck C, et al. MNE software for processing MEG and EEG data. Neuroimage. 2014;86:446–60. doi: 10.1016/j.neuroimage.2013.10.027 24161808 PMC3930851

[21] Welch P. The use of fast Fourier transform for the estimation of power spectra: A method based on time averaging over short, modified periodograms. IEEE Trans Audio Electroacoust. 1967;15(2):70–3. doi: 10.1109/tau.1967.1161901

[22] Liang K-Y, Zeger SL. Longitudinal data analysis using generalized linear models. Biometrika. 1986;73(1):13–22. doi: 10.1093/biomet/73.1.13

[23] Colin Cameron A, Miller DL. A practitioner’s guide to cluster-robust inference. J Hum Resour. 2015;50(2):317–72. doi: 10.3368/jhr.50.2.317

