# Supplementary material for: Frontal alpha asymmetry and instructed satisfaction-rating changes after price presentation: An exploratory pilot study
Source: PLoS One. 2026 Nov 2;21(11):e0361622. doi: 10.1371/journal.pone.0361622 (PMC13726993; doi:10.1371/journal.pone.0361622)
Supplement: Supplementary file 1 [file pone.0361622.s008.pdf]

S1 Appendix: Eligibility screening form

The items below were administered in person on a paper form by the first author at recruitment — at least 24 hours before the first experimental session and during the same contact when the participant information sheet was provided and written informed consent was obtained. All responses were based on participant self-report; no clinical interview, medical record, or documentary verification was used, and no diagnostic instrument was administered.

Responses were recorded as follows: age in years; annual personal income was recorded only as meeting or not meeting the stated ceiling, with no amount or band recorded; and all other items were recorded as yes/no responses. A participant was eligible only if all three inclusion items were met and all three exclusion items were answered “no”.

Participant record

Field	Recorded as
Participant code	de-identified code assigned at recruitment
Date of screening	date
Screening administered by	first author

Inclusion criteria

#	Item	Response
1	Age (years)	______ years · eligible if 18–27
2	Is your annual personal income below THB 360,000?	☐ Yes ☐ No · *(value not recorded)*
3	Do you have prior experience with online shopping?	☐ Yes ☐ No

Exclusion criteria

#	Item	Response
4	Do you have a history of neurological disorders — for example cerebrovascular disease, movement disorders, multiple sclerosis, or previous brain surgery?	☐ Yes ☐ No
5	Do you have a history of epilepsy or seizures?	☐ Yes ☐ No
6	Have you ever been diagnosed with a psychiatric condition that may affect cognition or emotional regulation?	☐ Yes ☐ No

Eligibility outcome

Eligible for the study	☐ Yes ☐ No
Participant information sheet provided	☐ Yes
Written informed consent obtained	☐ Yes · date: __________

**The form was administered in Thai; the wording above is a translation of the items, which restated the inclusion and exclusion criteria given in the Methods.*
